# Dynamic flavor formation and microbial succession during fermentation of water bamboo shoots: Insights from volatilomics and metagenomics

**DOI:** 10.1016/j.fochx.2026.104575

**Published:** 2026-10-12

**Authors:** Kai Zhu, Songheng Wu, Huilin Deng, Chenxia Liu, Jinglin Zhang, Chao Zhu, Yongjin Qiao, Yi Zhang

**Affiliations:** a Shanghai Academy of Agricultural Sciences, Shanghai 201403, China; b Key Laboratory of Germplasm Innovation and Genetic Improvement of Grain and Oil Crops (Co-construction by Ministry and Province), Ministry of Agriculture and Rural Affairs; Key Laboratory of Agricultural Genetics and Breeding of Shanghai, Crop Breeding and Cultivation Research Institute, Shanghai Academy of Agricultural Sciences; Shanghai 201403, China; c Faculty of Flavour Fragrance and Cosmetics, Shanghai Institute of Technology, Shanghai 201418, China; d Shanghai Jinshan Pickled Vegetables Seasoning Factory Co., Ltd, Shanghai 201500, China

**Keywords:** Fermented water bamboo shoot, Flavor compounds, Core microorganism, Correlation analysis, Metabolic pathway

## Abstract

This study investigated the dynamic changes in flavor compounds and microbial communities during fermented water bamboo shoot (FWBS) fermentation by integrating physicochemical analysis, electronic tongue, electronic nose, HS-SPME-GC–MS and metagenomics. These results showed that free amino acids exhibited an initial increase followed by a decrease, reaching a peak on day 5, while lactic acid accumulated rapidly as the predominant organic acid. *E*-tongue analysis classified FWBS fermentation into early (day1 – day 3), middle (day 5), and late stages (day 7 – day 9). A total of 76 VOCs were detected, mainly esters, phenols and alcohols. Spearman correlation analysis indicated that *Lactiplantibacillus*, *Lactococcus*, *Leuconostoc*, *Weissella* were positively correlated with 14 key VOCs (VIP > 1, OAV > 1, and *P* < 0.05) These findings provide new insights into the flavor formation mechanism of FWBS and establish a foundation for quality control and targeted fermentation regulation.

## Highlights

• 14 key VOCs with OAV > 1 and VIP > 1 were identified.
• Microbial community succession was systematically characterized
• Core microorganisms closely associated with key flavor compounds were revealed.
• Potential metabolic pathways involved in flavor formation were reconstructed.

## 1 Introduction

Water bamboo shoot (*Zizania latifolia*), commonly known as “jiaobai” in China, is a perennial aquatic herb widely cultivated and consumed in East Asian. Its edible swollen stem, formed after infection by *Ustilago esculenta*, is appreciated for the crisp texture and distinctive flavor (Liu et al., 2026). Additionally, water bamboo shoot (WBS) is rich in dietary fiber, proteins, vitamins, minerals, and diverse bioactive compounds, which confer antioxidant, anti-inflammatory, hypoglycemic, and lipid-lowering activities (Gao et al., 2022). WBS has a cultivation area of more than 20,000 ha in Zhejiang Province, with an annual fresh production of approximately 500,000 tons (Chen et al., 2022). However, fresh WBS is highly susceptible to postharvest deterioration, such as browning, water loss, lignification, and texture softening, which greatly limits its storage stability and commercial circulation (Qi et al., 2020). Therefore, a promising strategy is needed to reduce losses and promote the value-added utilization of WBS.

Vegetable fermentation is a traditional technique that not only prolongs shelf life, but also improves sensory quality and nutritional property (Luo et al., 2023). Microbial metabolism drives the conversion of carbohydrates, proteins, and other endogenous substrates into various metabolites throughout fermentation, thereby shaping the characteristic taste and aroma of the final product (Peng et al., 2025). Among these metabolites, organic acids (OAs) are the main contributors to sourness and acidification, while free amino acids (FAAs) play important roles in taste formation, especially umami, sweetness, and bitterness, and also serve as precursors for aroma compounds (Xiong et al., 2024). Previous studies have shown that *Zizania latifolia* contains 16 FAAs, and its composition is significantly affected by cultivar and harvest season (Hu et al., 2023). OAs metabolism has been implicated in postharvest quality changes in *Zizania latifolia*, suggesting its relevance to aroma formation (Bata Gouda et al., 2021). We previously compared the effects of different drying methods on the volatile profiles of dried WBS using GC-IMS and identified 52 VOCs, among which 3-methyl-butanal and 5-methyl-2-furfural were kye characteristic VOCs (Tong et al., 2025). However, few studies have systematically characterized the dynamic evolution of OAs, FAAs, and VOCs throughout FWBS fermentation. More importantly, microbial succession may reshape substrate utilization and community metabolic potential during fermentation, thereby influencing the formation of different classes of flavor compounds, particularly esters and phenolic compounds (Xiong et al., 2024). Multiple metabolic pathways, including carbohydrate, amino acid, and fatty acid metabolism, may further contribute differently to flavor formation through the generation and transformation of flavor precursors and volatile metabolites (Xiong et al., 2024). Nevertheless, the mechanistic links between microbial succession and the formation of specific volatile classes, as well as the respective roles of these metabolic pathways in shaping the overall flavor profile of FWBS, remain insufficiently understood.

With growing interest in flavor science, multidimensional flavor characterization has become an effective approach for elucidating quality formation in fermented foods (Ruan et al., 2026). Recently, electronic nose and electronic tongue, have been increasingly used as rapid and non-destructive tools for evaluating food quality and sensory characteristics. Xu et al. (2021) found that electronic nose sensor responses, combined with a swarm clustering algorithm, could effectively classify the freshness levels of *Zizania latifolia* during a 20-day storage period, and the classification results were consistent with changes in sensory quality, hardness, and pH. Moreover, the study by Wei et al. (2025b) found that electronic tongue analysis combined with sensory evaluation and non-targeted metabolomics could reveal the taste evolution of pickled bamboo shoots during fermentation, with amino acids, organic acids, peptides, flavonoids, sugars, and other metabolites contributing to changes in taste characteristics. Microbial succession is widely recognized as the major driving force governing flavor formation and quality stability in fermented vegetables. In traditional fermented vegetables such as kimchi, suancai, pickled mustard, lactic acid bacteria (LAB) usually become dominant during fermentation and play essential roles in acidification, substrate conversion, and flavor generation (Leroy & De Vuyst, 2004). Besides, significant correlations between specific microbial taxa and key flavor compounds have been reported in several fermented vegetables. Liao et al. (2023) found that *Leuconostoc*, *Weissella*, *Lactobacillus*, *Candida*, *Pichia*, and *Kazachstania* were closely associated with spicy, fruity, and floral aroma formation during chili fermentation. In fermented bamboo shoots, the accumulation of lactic and acetic acids promoted microbial succession from acid-sensitive genera, such as *Lactococcus*, *Leuconostoc*, and *Weissella* (Guan et al., 2022). Accordingly, the identification of core functional microorganisms and key flavor substances is considered fundamental for the targeted regulation of product quality and the development of starter cultures.

High-throughput sequencing has greatly advanced the understanding of microbial ecology in fermented foods (Wei & Huang, 2025). Compared with culture-dependent methods and 16S rRNA amplicon sequencing, metagenomic sequencing could provide higher taxonomic resolution and enable the characterization of functional genes and metabolic pathways in complex fermentation ecosystems (Wei et al., 2025). Recent studies have shown that fermented microbiota is strongly shaped by raw materials and fermentation conditions and is closely associated with flavor formation, quality attributes, and safety (Caffrey et al., 2024; Miao et al., 2025). Therefore, metagenomics is a powerful tool for identifying core microorganisms and key metabolic functions, providing a theoretical basis for starter culture development and fermentation quality control. Previous studies on fermented water bamboo shoot (FWBS) have mainly focused on fermentation process optimization, salt concentration regulation, and the screening of lactic acid bacteria to improve product quality and fermentation efficiency. Nevertheless, most of these studies emphasized product quality improvement rather than flavor formation mechanisms, and the succession of microbial communities and their interactions with flavor formation have not been systematically elucidated.

In this study, the dynamic variations in OAs, FAAs, and VOCs during FWBS fermentation were systematically examined. In addition, electronic nose and electronic tongue analyses were performed to comprehensively evaluate the flavor profile, and metagenomic sequencing was applied to characterize the microbial diversity and succession patterns. Furthermore, key characteristic VOCs were screened by multivariate statistical analysis, and the core microorganisms closely associated with flavor formation were identified through spearman correlation analysis. This study provides a scientific basis for elucidating the flavor formation mechanism of FWBS, and lays the foundation for functional starter screening, inoculated fermentation, and the development of FWBS products.

## 2 Materials and methods

### 2.1 Preparation and sampling

Fresh water bamboo shoots (*Zizania latifolia*), cultivar “Hangzhou”, were obtained from a commercial farm in Qingpu District, Shanghai, China (31°15′ N, 121°12′ E; altitude 3.0 m). After harvest, the samples were immediately transported to the laboratory and stored at 4 °C. Prior to fermentation, the samples were washed to remove surface impurities, cut into uniform pieces (3 cm × 2 cm × 2 cm, length × width × height), and air-dried at 25 °C until the moisture content decreased to 70%. Subsequently, the pretreated samples were mixed with 6% (*w*/w) salt and pickled. Fermented samples were collected after 1, 3, 5, 7, and 9 days of pickling. At each sampling point, the samples were immediately sealed and divided into two portions. One portion was stored at −20 °C for flavor components analysis, while the other portion was stored at −80 °C for microbial diversity analysis.

### 2.2 Determination of physicochemical properties

The pH was determined using a PHS-3C pH meter (Shanghai INESA Scientific Instrument Co., Ltd., Shanghai, China). Titratable acidity was determined by potentiometric titration with 0.1 mol/L NaOH solution. Briefly, 10.0 g of sample was homogenized with 90 mL of distilled water and filtered, the filtrate was titrated with standardized 0.1 mol/L NaOH solution until the pH reached 8.2, as determined using a PHS-3C pH meter (Shanghai INESA Scientific Instrument Co., Ltd., Shanghai, China). Titratable acidity was expressed as lactic acid equivalent. The texture (hardness, springiness) was examined according to the approach described by Chen et al. (2023).

### 2.3 Determination of free amino acids (FAAs)

Free amino acids (FAAs) in fermented water bamboo shoots were determined based on the previously reported method with slight modifications (Asimi & Min, 2025). Briefly, 1 g of sample was mixed with 1 mL of 0.1 M HCl and extracted at 110 °C for 1 h. The extract was filtered through a 0.22 μm membrane filter. Then, 10 μL of the filtrate was mixed with 70 μL of borate buffer and 20 μL of AccQ•Tag reagent in a UPLC vial. The mixture was equilibrated at 25 °C for 1 min and derivatized at 55 °C for 10 min. Subsequently, 1 μL of the derivatized solution was injected for analysis. Separation was performed on an ACQUITY UPLC BEH C18 column (2.1 × 50 mm, 1.7 μm) coupled with a Q Exactive hybrid quadrupole–Orbitrap mass spectrometer equipped with a heated electrospray ionization source (Thermo Fisher Scientific, MA, USA). The column temperature was maintained at 55 °C, and the flow rate was 0.5 mL/min. The mobile phases were 0.1% formic acid in water (A) and acetonitrile (B). The gradient program was set as follows: 95:5 (A: B, *v*/v) at 0 min, 90:10 at 5.5 min, 75:25 at 7.5 min, 40:60 at 8.0 min, and 95:5 at 8.5–13.0 min. Data acquisition and processing were performed using Xcalibur 4.1 and TraceFinder™ 4.1 Clinical software, respectively.

### 2.4 Determination of organic acids (OAs)

Organic acids (OAs) in fermented water bamboo shoots were extracted and analyzed according to the previous methods with slight modifications (Chen et al., 2024). Briefly, 1 g of sample was extracted with 1 mL of methanol/chloroform (7,3, *v*/v) in an ice bath for 30 min. After adding 600 μL of water, the mixture was centrifuged at 12,000 rpm for 10 min at 4 °C. The supernatant was collected, and the extraction was repeated once. The combined supernatants were derivatized with 10 μL of 0.1 M EDC and 10 μL of 0.1 M 3-NPH at 40 °C for 30 min. The derivatized extracts were analyzed using a Vanquish UPLC system coupled with a Q Exactive hybrid quadrupole–Orbitrap mass spectrometer (Thermo Fisher Scientific, Bremen, Germany). Separation was performed on a Waters BEH C18 column (50 × 2.1 mm, 1.8 μm) at 40 °C. The mobile phases were 0.1% formic acid in water (A) and acetonitrile (B), with a flow rate of 0.35 mL/min and an injection volume of 2 μL. The gradient program was as follows: 90:10 (A:B, *v*/v) at 0–2.0 min, 10:90 at 12.0–14.0 min, and 90:10 at 14.1–16.0 min. Mass spectrometric data were acquired in Full MS/dd-MS2 mode using the following parameters: spray voltage, −2.8 kV; sheath gas, 40 arb; auxiliary gas, 10 arb; capillary temperature, 320 °C; and auxiliary gas heater temperature, 350 °C. Data acquisition and processing were performed as described for FAA analysis.

### 2.5 Electronic tongue (*E*-tongue) analysis

The taste profile was evaluated using an electronic tongue system (Insent, SA-402B, Tokyo, Japan) based on a previously reported method with slight modifications (Wei et al., 2025). Briefly, the homogenized sample was mixed with ultrapure water at a ratio of 1:10 (*w*/*v*) and homogenized at 8000 r/min for 1 min. The mixture was then incubated in a water bath at 50 °C for 20 min, followed by centrifugation at 5000 r/min for 10 min. The supernatant was collected and filtered prior to analysis. Before measurement, the sensors were sequentially cleaned in cleaning solution for 90 s and in reference solution for 120 s twice, followed by stabilization at the reference position for 30 s. During measurement, the sample was analyzed for 30 s to obtain the initial taste values. Subsequently, the sensors were rinsed with reference solution for 3 s and immersed in a fresh reference solution for 30 s to record the aftertaste response. Five taste sensors (C00, AE1, CA0, CT0, and AAE) were used for the analysis. Each sample was tested for four consecutive cycles. To reduce the influence of system drift, the first cycle was excluded, and the mean of the remaining three cycles was recorded as the final result.

### 2.6 Electronic nose (*E*-nose) analysis

The volatile odor profile of fermented samples was determined using a PEN3 electronic nose system (Airsense Analytics GmbH, Schwerin, Germany), which was equipped with ten metal oxide semiconductor sensors. The analysis was performed according to previously reported methods with minor modifications (Lee et al., 2025). Briefly, 3 g of sample was placed into a 15 mL headspace vial, immediately sealed with a PTFE-coated cap, and equilibrated at 40 °C for 30 min to allow sufficient enrichment of volatile compounds. The headspace gas was then introduced into the sensor chamber through the sampling needle for signal acquisition. The sampling flow rate was set at 150 mL/min, and the detection time was 120 s. After each measurement, the system was purged with filtered air for 120 s to restore the baseline before the next injection. Each sample was analyzed in quintuplicate. The sensor response values obtained at a stable acquisition period were used for subsequent statistical analysis. The main sensitive substances corresponding to the ten sensors are listed in Table S1.

### 2.7 Determination of volatile organic compounds (VOCs)

Volatile organic compounds (VOCs) were characterized using HS-SPME-GC–MS based on a modified procedure described by Zhang et al. (2024). A DB-WAX column (30 m × 0.25 mm × 0.25 μm, Agilent Technologies, USA) was applied on analyte separation. Before extraction, the SPME fiber was activated in the GC injector at 240 °C for 1 h. Then, 5 mL of sample was transferred into a 20 mL headspace vial, followed by the addition of 1 g NaCl to improve volatile release. Finally, 10 μL of deuterated n-hexanol - d13 internal standard solution (0.05 mg/mL) was added. The SPME device was inserted approximately 1 cm into the vial headspace, and the fiber was exposed for extraction. Volatile adsorption was performed at 40 °C for 30 min under thermostatic conditions. After extraction, the fiber was immediately introduced into the GC injection port and desorbed at 240 °C for 5 min for GC–MS analysis. The GC oven program was set as follows: the initial temperature was maintained at 40 °C for 3 min, increased to 170 °C at 5 °C/min, and then raised to 230 °C at 8 °C/min. Helium with a purity of 99.99% was used as the carrier gas at a constant flow rate of 1 mL/min. The MS parameters were as follows: electron ionization mode, ion source temperature of 230 °C, and mass scan range of 35–500 amu. Volatile compounds in the samples were identified by matching their mass spectra with the NIST library and comparing their linear retention indices (LRIs). The LRIs were calculated using the retention times of n-alkane standards (C7–C30). Semi-quantitative analysis was performed using cyclohexanone as the internal standard, and the relative concentrations of volatile compounds were calculated according to Eq.

$$C_{X}=\frac{C_{1}\times V_{1}\times S_{x}}{S_{1}\times V_{0}}$$

Where *C*_1_ is the concentration of the internal standard; *V*_1_ is the volume of the internal standard; *S*_x_ is the peak area of volatile compounds, *C*_x_ is the concentration of volatile compounds; *S*_1_ is the peak area of the internal standard; *V*_0_ is the volume of the samples.

### 2.8 Calculation of odor activity values

OAV analysis was used to evaluate the aroma contribution of volatile compounds. The OAV of each compound was calculated as the ratio of its measured concentration to the corresponding odor threshold in water. The threshold values were obtained from previously reported references (Ong & Acree, 1999; Powers & Quinlan, 1973; Schnabel et al., 1988; Seideneck & Schieberle, 2011; Van Gemert, 2011). Compounds with OAVs ≥1 were considered aroma-active compounds contributing to the sample aroma profile (Feng et al., 2018).

### 2.9 Analysis of metagenomic

To investigate microbial community succession during water bamboo shoot fermentation, metagenomic sequencing was conducted according to previously described methods with minor modifications (Peng et al., 2025). Total microbial DNA was extracted from fermented samples using a magnetic bead-based DNA extraction kit according to the manufacturer's protocol. The quality and integrity of the extracted DNA were evaluated by 1% agarose gel electrophoresis, and DNA concentration was quantified using a Qubit® dsDNA Assay Kit with a Qubit® 2.0 Fluorometer.

High-quality DNA samples were used for library construction. Briefly, 1 μg of genomic DNA was sheared into 350 bp fragments using a Covaris ultrasonic shearing system. Metagenomic libraries were prepared using the NEBNext® Ultra DNA Library Prep Kit for Illumina following the manufacturer's instructions, including end repair, A-tailing, adapter ligation, purification, and PCR enrichment. The constructed libraries were quantified using a Qubit® 2.0 Fluorometer, and their insert sizes were examined using an Agilent 2100 Bioanalyzer. The effective library concentration was accurately determined by qPCR, and libraries with concentrations greater than 3 nM were selected for sequencing. Qualified libraries were pooled and sequenced on an Illumina platform using a paired-end 150 bp strategy.

### 2.10 Bioinformatic analysis

After sequencing, raw reads were processed to eliminate adapter sequences, low-quality reads, and reads containing ambiguous nucleotides. The clean reads were assembled using MEGAHIT (v1.1.3). Clean reads from each sample were mapped to the non-redundant gene catalog using SOAPAligner (v2.21) to estimate gene abundance. For taxonomic annotation, the representative sequences of the non-redundant gene catalog were aligned against the NCBI Non-Redundant Protein (NR) database using DIAMOND, and taxonomic abundance was calculated based on the abundance of the annotated genes. Functional annotation was performed against the Kyoto Encyclopedia of Genes and Genomes (KEGG) database using BLAST+ (v2.2.31+) with an *E*-value cutoff of 1 × 10^−5^. KEGG Orthology (KO), enzyme commission (EC), and pathway annotations were subsequently used to characterize the functional profiles of the microbial communities. Microbial alpha-diversity, represented by observed OTUs, Shannon, Simpson, Chao1, and Good's coverage indices, and beta-diversity based on Bray–Curtis dissimilarity were analyzed using QIIME 2 (version 2020.6). LEfSe analysis was further conducted to screen microbial taxa that significantly differentiated fermentation stages. Other figures were produced using R packages (version 3.4.4).

### 2.11 Statistical analysis

To ensure experimental reproducibility, all measurements were performed in triplicate. Differences among samples were evaluated using one-way ANOVA followed by Duncan's multiple range test in SPSS Statistics 22.0 (SPSS Inc., Chicago, IL, USA), with significance levels set at *P* < 0.05 and *P* < 0.01. Graphs of basic physicochemical properties, organic acids, and free amino acids were generated using OriginPro 2024 software (Origin Lab Corporation, USA). Multivariate statistical analyses, including principal component analysis (PCA), hierarchical cluster analysis (HCA), partial least squares discriminant analysis (PLS-DA), model validation with permutation testing (*n* = 200), and variable importance in projection (VIP) analysis were conducted using SIMCA software (Version 14.1). Spearman correlation analysis between microorganisms and volatile flavor compounds was performed using R software (version 4.3.1) with appropriate packages (heatmap, scatterpie, reshape2).

## 3 Results and discussion

### 3.1 Physicochemical analysis

Physicochemical properties are important indicators for evaluating the fermentation process and quality changes of fermented vegetables (Li, Ma, et al., 2026). As shown in Fig. 1A, the pH of FWBS gradually decreased during fermentation, whereas titratable acidity showed an opposite increasing trend. The pH decreased from 4.45 on day 1 to 3.64 on day 9, while titratable acidity increased from 0.28 g/100 g to 0.86 g/100 g. A lower pH could inhibit the growth of spoilage microorganisms, thereby improving the microbial safety and storage stability of fermented vegetables (Liu et al., 2024). Meanwhile, fermented pickles are considered to reach a mature when the pH below 4.0 and the titratable acidity exceeds 0.3 g/100 g (Tang et al., 2022). As shown in Fig. 1B, both hardness and springiness of FWBS decreased gradually during fermentation. Hardness declined from 2850 g on day 1 to 1760 g on day 9, while springiness decreased from 0.92 to 0.72. This result suggested that the texture of FWBS gradually softened as fermentation progressed. The decrease in hardness and springiness may be related to the destruction of plant tissue structure during fermentation. The acidic environment may weaken cell wall integrity, while endogenous and microbial enzymes may promote the degradation of cell wall components, such as pectin, cellulose and hemicellulose (Li, Yang, et al., 2026).

**Fig. 1:**
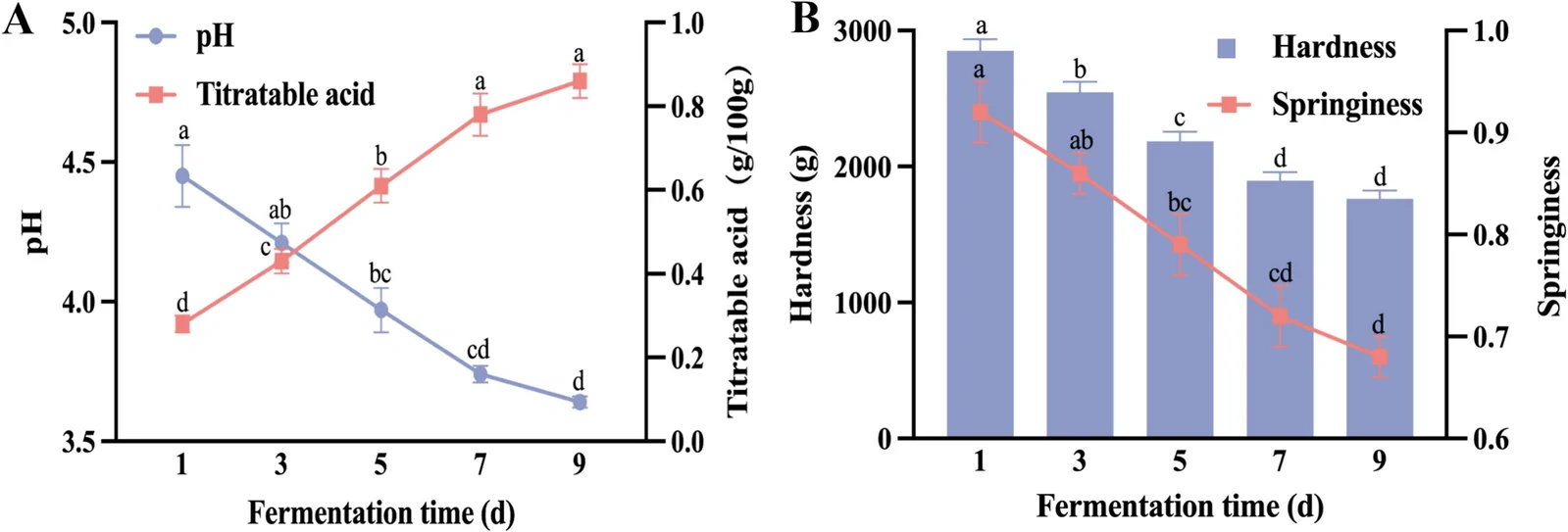
Changes in physicochemical properties during water bamboo shoot fermentation. (A) pH and titratable acidity. (B) Hardness and springiness.

### 3.2 Free amino acids (FAAs) analysis

Free amino acids (FAAs) are key non-volatile flavor compounds in fermented vegetables, directly contributing to taste and serving as precursors for volatile aroma compounds through microbial metabolism (Peng et al., 2025). A total of 20 FAAs were detected in FWBS and grouped into umami amino acids (Asp and Glu), sweet amino acids (Ser, Gly, Thr, Ala, and Pro), bitter amino acids (His, Arg, Cit, Lys, Tyr, Met, Val, Ile, Leu, Phe, and Trp), and tasteless amino acids (GABA and Cys) according to the taste characteristics (Table 1). During the fermentation of FWBS, the total FAAs content first increased and then decreased, rising from day 1 (322.75 μg/g) to the highest on day 5 (499.23 μg/g), and then gradually decreasing to day 9 (374.96 μg/g). This trend was similar to that observed in other fermented vegetables (Chen et al., 2024; Zhang et al., 2024), which may be attributed to the dynamic balance between protein hydrolysis and microbial utilization. During early fermentation, tissue disruption and enzymatic hydrolysis promoted the breakdown of proteins and peptides, leading to FAA accumulation (Hu et al., 2025). As fermentation progressed, FAAs were further utilized by dominant microorganisms, particularly LAB, as nitrogen sources or converted into flavor-related metabolites, resulting in the decrease (Chen et al., 2024). Additionally, the taste FFAs showed a trend similar to that of total FFAs, with their relative abundance following the order of bitter amino acids (BAAs) > sweet amino acids (SAAs) > umami amino acids (UAAs). Asp and Glu, the major UAAs, increased continuously during fermentation and peaked on day 7, indicating an enhancement of umami taste in the middle and late fermented stages (day 7 – day 9). SAAs increased in the early fermented stage (day1 – day 3), contributing to taste softness and palatability. Bitter amino acids were the predominant taste FAAs and reached the highest level on day 5. Notably, GABA was the predominant individual FAAs throughout fermentation, increasing from 153.595 μg/g on day 1 to 212.862 μg/g on day 5. As a bioactive non-protein amino acid, GABA has attracted increasing attention in fermented foods because of its potential health-related functions, including stress regulation, sleep improvement, and blood pressure modulation (Aoki et al., 2002). Its accumulation is generally associated with microbial glutamate decarboxylation, suggesting that glutamate-related metabolism may be active during FWBS fermentation. Glu could be converted into GABA via glutamate decarboxylase-mediated decarboxylation during microbial fermentation. Therefore, the increased GABA level in FWBS may be attributed to the active conversion of Glu during fermentation (Zhang et al., 2026).

**Table 1:** Changes in free amino acid content during the fermentation of water bamboo shoots.

Taste attributes	Content (ug/g)	Fermentation time (d)				
		Day 1	Day 3	Day 5	Day 7	Day 9
Umami	Asp	2.404 ± 0.056 ^e^	3.895 ± 0.023 ^d^	6.711 ± 0.143 ^c^	12.076 ± 0.153 ^a^	7.921 ± 0.037 ^b^
	Glu	3.536 ± 0.063 ^e^	4.866 ± 0.046 ^d^	6.761 ± 0.189 ^c^	9.384 ± 0.083 ^a^	7.836 ± 0.115 ^b^
	Umami amino acids	5.94 ± 0.12 ^e^	8.76 ± 0.06 ^d^	13.47 ± 0.33 ^c^	21.46 ± 0.22 ^a^	15.76 ± 0.14 ^b^
Sweet	Ser	7.327 ± 0.117 ^a^	7.226 ± 0.123 ^a^	4.324 ± 0.104 ^b^	3.364 ± 0.030 ^c^	3.111 ± 0.053 ^d^
	Gly	14.684 ± 0.227 ^c^	15.593 ± 0.215 ^b^	17.747 ± 0.463 ^a^	17.220 ± 0.173 ^a^	14.140 ± 0.139 ^c^
	Thr	11.416 ± 0.313 ^c^	12.966 ± 0.170 ^b^	15.457 ± 0.322 ^a^	14.974 ± 0.139 ^a^	12.541 ± 0.219 ^b^
	Ala	18.896 ± 0.071 ^d^	21.334 ± 0.400 ^c^	27.311 ± 0.347 ^a^	27.948 ± 0.242 ^a^	26.000 ± 0.108 ^b^
	Pro	14.574 ± 0.170 ^d^	16.267 ± 0.238 ^c^	19.263 ± 0.257 ^b^	21.065 ± 0.111 ^a^	15.797 ± 0.138 ^c^
	Sweet amino acids	66.90 ± 0.16 ^c^	73.39 ± 1.14 ^b^	84.10 ± 1.46 ^a^	84.57 ± 0.50 ^a^	71.59 ± 0.42 ^b^
Bitter	His	14.756 ± 0.645 ^c^	12.516 ± 0.177 ^d^	19.324 ± 0.306 ^a^	15.730 ± 0.244 ^b^	12.509 ± 0.034 ^d^
	Arg	2.449 ± 0.085 ^d^	3.201 ± 0.094 ^c^	8.595 ± 0.167 ^a^	7.262 ± 0.083 ^b^	3.231 ± 0.059 ^c^
	Cit	0.735 ± 0.010 ^e^	1.437 ± 0.024 ^d^	1.827 ± 0.062 ^b^	2.008 ± 0.030 ^a^	1.577 ± 0.010 ^c^
	Lys	2.424 ± 0.425 ^d^	5.901 ± 0.199 ^c^	13.187 ± 0.274 ^a^	12.781 ± 0.099 ^a^	9.526 ± 0.064 ^b^
	Tyr	17.604 ± 0.844 ^c^	23.006 ± 0.301 ^b^	28.472 ± 0.650 ^a^	14.291 ± 0.137 ^d^	8.994 ± 0.048 ^e^
	Met	1.766 ± 0.026 ^d^	3.564 ± 0.063 ^c^	6.335 ± 0.187 ^b^	8.295 ± 0.099 ^a^	6.157 ± 0.091 ^b^
	Val	11.234 ± 0.720 ^c^	19.122 ± 0.286 ^b^	22.898 ± 0.368 ^a^	23.380 ± 0.319 ^a^	20.242 ± 0.178 ^b^
	Ile	7.546 ± 0.255 ^e^	9.891 ± 0.142 ^d^	13.359 ± 0.376 ^b^	14.082 ± 0.199 ^a^	11.944 ± 0.110 ^c^
	Leu	16.992 ± 0.386 ^e^	21.157 ± 0.229 ^d^	32.398 ± 0.705 ^b^	36.735 ± 0.476 ^a^	29.871 ± 0.229 ^c^
	Phe	16.892 ± 0.503 ^d^	22.677 ± 0.357 ^c^	31.185 ± 0.690 ^a^	30.455 ± 0.423 ^a^	27.520 ± 0.197 ^b^
	Trp	3.579 ± 0.090 ^d^	6.151 ± 0.050 ^c^	10.238 ± 0.227 ^a^	10.370 ± 0.112 ^a^	8.470 ± 0.052 ^b^
	Bitter amino acids	95.98 ± 3.06 ^e^	128.62 ± 1.86 ^d^	187.82 ± 3.96 ^a^	175.39 ± 1.92 ^b^	140.04 ± 0.97 ^c^
	GABA	153.595 ± 1.933 ^d^	183.714 ± 2.903 ^b^	212.862 ± 4.421 ^a^	167.509 ± 2.282 ^c^	146.728 ± 2.186 ^d^


Taste attributes	Content (ug/g)	Fermentation time (d)				
		Day 1	Day 3	Day 5	Day 7	Day 9
	Cys	0.343 ± 0.007 ^e^	1.455 ± 0.032 ^a^	0.978 ± 0.009 ^c^	1.105 ± 0.012 ^b^	0.846 ± 0.008 ^d^
	Tasteless amino acids	153.94 ± 1.93 ^d^	185.17 ± 2.92 ^b^	213.84 ± 4.43 ^a^	168.61 ± 2.29 ^c^	147.57 ± 2.19 ^d^
	Total amino acids	322.75 ± 1.89 ^e^	395.94 ± 5.93 ^c^	499.23 ± 10.15 ^a^	450.03 ± 4.53 ^b^	374.96 ± 3.62 ^d^

Different superscript letters in the same row indicate significantly difference (*P* < 0.05).

### 3.3 Organic acids (OAs) analysis

Organic acids (OAs) are also important non-volatile flavor compounds in fermented vegetables and play a crucial role in sour taste formation (Zhu, Wu, et al., 2024). As shown in Table 2, A total of 10 OAs were detected during the fermentation of WBS, including lactic acid, propionic acid, tartaric acid, butyric acid, malic acid, succinic acid, isovaleric acid, fumaric acid, oxalic acid, and citric acid. At the initial stage of fermentation (day 1 – day 3), oxalic acid and malic acid were the predominant OAs. Oxalic acid exhibited the highest concentration on day 1, reaching 5312.037 mg/kg, but showed an overall decreasing trend during fermentation, declining to 1594.964 mg/kg by day 9. Similarly, malic acid decreased markedly from 3682.573 mg/kg on day 1 to 25.597 mg/kg on day 9. Similar decreasing trends were observed for citric acid and fumaric acid, which declined from 461.088 and 160.275 mg/kg to 7.648 and 1.235 mg/kg, respectively. In contrast, lactic acid increased rapidly and became the predominant organic acid during fermentation. Its concentration increased from 1794.010 mg/kg on day 1 to 13,276.960 mg/kg on day 3 and reached the highest level of 13,677.385 mg/kg on day 5. The accumulation of lactic acid could provide a mild and characteristic sourness, mask undesirable tastes, and contribute to pH reduction and microbial inhibition in FWBS (Xiong et al., 2024). Succinic acid increasing from day 1 (1657.550 mg/kg) to day 3 (7638.416 mg/kg), and maintaining a relatively high level throughout fermentation, which generally associated with sour, salty, and umami-like taste. Meanwhile, although propionic acid, butyric acid, and isovaleric acid were at relatively low concentrations, they may still enhance the fermented aroma and flavor complexity of FWBS due to their low odor thresholds and potential synergistic effects (Zhu, Deng, et al., 2024).

**Table 2:** Changes in organic acid content during the fermentation of water bamboo shoots.

Content (mg/kg)	Fermentation time (d)				
	Day 1	Day 3	Day 5	Day 7	Day 9
Lactic acid	1794.010 ± 11.185 ^e^	13,276.960 ± 199.274 ^b^	13,677.385 ± 107.447 ^a^	6302.291 ± 130.791 ^d^	9396.229 ± 173.488 ^c^
Propionic acid	19.833 ± 0.131 ^c^	21.663 ± 0.167 ^b^	20.852 ± 0.178 ^b^	36.841 ± 0.695 ^a^	20.718 ± 0.369 ^bc^
Tartaric acid	0.361 ± 0.010 ^a^	0.155 ± 0.006 ^c^	0.129 ± 0.004 ^d^	0.103 ± 0.004 ^e^	0.184 ± 0.006 ^b^
Butyric acid	3.324 ± 0.118 ^c^	3.961 ± 0.093 ^b^	3.932 ± 0.049 ^b^	4.672 ± 0.173 ^a^	3.853 ± 0.193 ^b^
Malic acid	3682.573 ± 16.922 ^a^	610.711 ± 9.152 ^b^	31.151 ± 0.650 ^c^	8.268 ± 0.240 ^c^	25.597 ± 0.417 ^c^
Succinic acid	1657.550 ± 14.859 ^e^	7638.416 ± 116.128 ^a^	6881.653 ± 73.098 ^b^	3319.062 ± 65.565 ^d^	5907.880 ± 155.723 ^c^
Isovaleric acid	4.158 ± 0.070 ^a^	3.282 ± 0.060 ^bc^	3.505 ± 0.022 ^b^	3.127 ± 0.103 ^c^	3.879 ± 0.240 ^a^
Fumaric acid	160.275 ± 2.313 ^a^	50.270 ± 2.307 ^b^	2.288 ± 0.115 ^c^	2.161 ± 0.013 ^c^	1.235 ± 0.039 ^c^
Oxalic acid	5312.037 ± 14.198 ^a^	4856.920 ± 114.653 ^b^	2527.648 ± 10.153 ^d^	3666.398 ± 9.287 ^c^	1594.964 ± 23.484 ^e^
Citric acid	461.088 ± 1.965 ^a^	184.279 ± 5.805 ^b^	8.771 ± 0.933 ^c^	2.176 ± 0.069 ^c^	7.648 ± 0.371 ^c^

Different superscript letters in the same row indicate significantly difference (*P* < 0.05).

### 3.4 *E*-tongue analysis

Electronic tongue analysis could better distinguish the taste characteristics of fermented vegetable at different fermentation stages and provide an overall evaluation of their non-volatile flavor profiles (Wei et al., 2025). As shown in Fig. 2A, Sourness, bitterness, astringency, aftertaste, umami, richness, and saltiness of FWBS all changed markedly during fermentation. The sourness response reached the lowest value on day 5, which was consistent with the high accumulation of lactic acid at this stage (Table 2). PCA confirmed the differentiation of taste profiles among FWBS samples at different fermentation stages (Fig. 2B). PC1 and PC2 explained 56.4% and 21.5% of the total variance, respectively, with a cumulative contribution of 77.9%. This indicated that the first two PCA effectively represented the overall taste information of the FWBS samples. Moreover, no overlap was observed among the samples, indicating that the taste characteristics of FWBS were clearly differentiated at different fermentation stages (Wei & Huang, 2025). The loading plot of electronic tongue analysis was used to further clarify the contribution of different taste attributes to sample discrimination. As shown in Fig. 2C, umami, saltiness, astringency, aftertaste-A, and richness were mainly located on the positive side of PC1, whereas sourness, bitterness and aftertaste-B were distributed on the negative side, indicating their opposite contributions to sample discrimination. Overall, the *E*-tongue analyses effectively discriminated different fermentation stages of FWBS, categorizing the fermentation process into early (day 1–3), middle (day 5), and late stages (day 7–9).

**Fig. 2:**
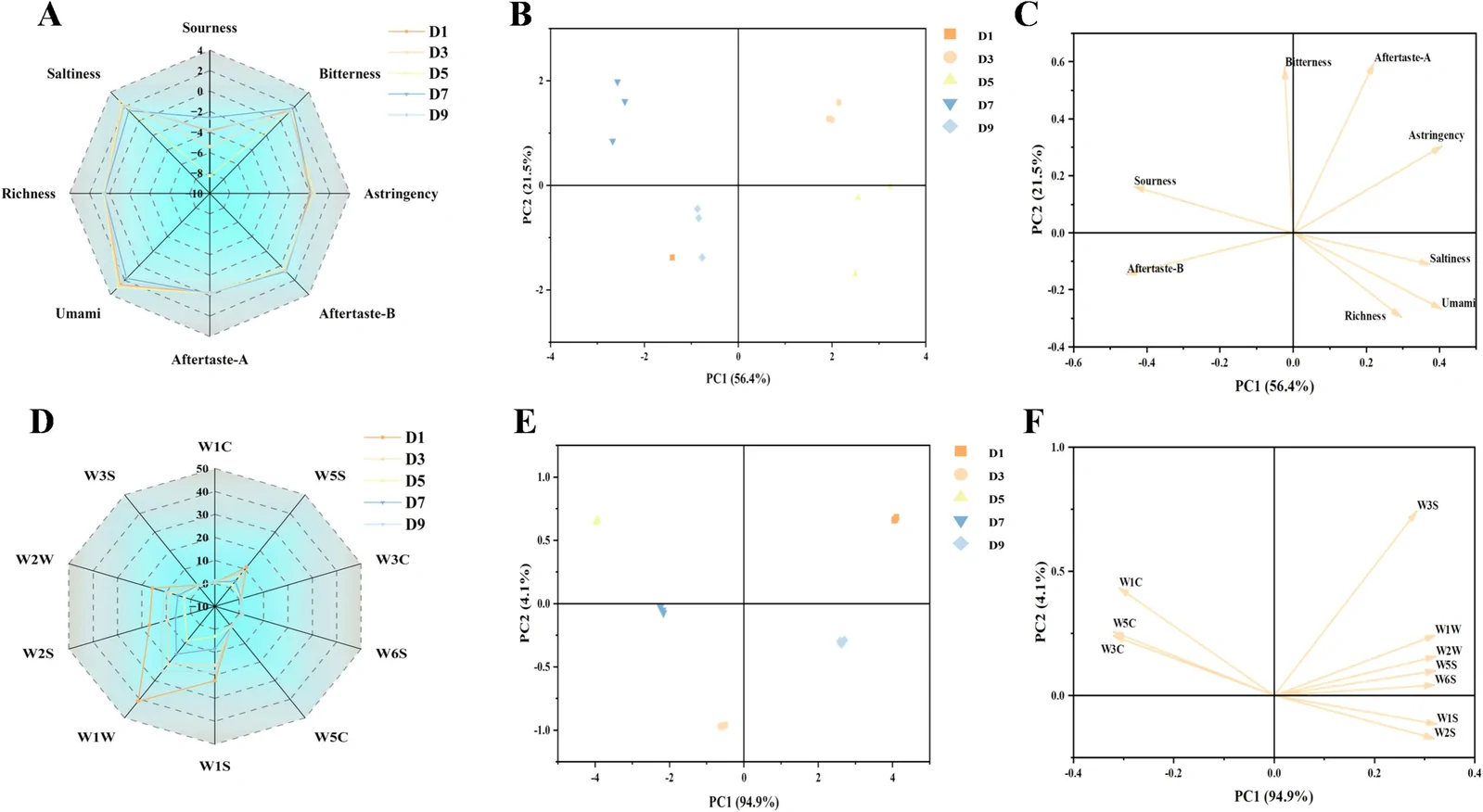
Electronic tongue and electronic nose analyses during water bamboo shoot fermentation. (A, D) Radar plots, (B, E) PCA score plots, (C, F) PCA loading plots of electronic nose sensor responses and electronic tongue taste attributes. D1, D3, D5, D7 and D9 represent fermentation days 1, 3, 5, 7 and 9, respectively.

### 3.5 E-nose analysis

As another intelligent sensory analysis technique, the electronic nose (E-nose) was used to characterize the volatile odor profiles of FWBS during fermentation (Lee et al., 2025). As shown in Fig. 2D, the E-nose consisted of 10 metal oxide sensors with different sensitivities to volatile compounds, including W1C, W5S, W3C, W6S, W5C, W1S, W1W, W2S, W2W, and W3S. W1W, W1S, W2S, W2W, and W5S showed strong response intensities and clear variations among FWBS samples, indicating that these sensors were effective in distinguishing samples from different fermentation stages. These results suggested that alcohols, aldehydes, ketones, methane-like compounds, sulfur-containing compounds, aromatic compounds, and nitrogen oxides were closely involved in the aroma evolution of FWBS during fermentation. As shown in Fig. 2E, PC1 explained 94.98% of the total variance and was the main contributor to the separation of FWBS aroma profiles. The loading plot indicated that W1W, W2W, W2S, W1S, W3S, and W5S were positively loaded on PC1, indicating that these sensors contributed greatly to the differentiation of FWBS aroma profiles (Fig. 2F). These results indicated that the aroma profile of FWBS gradually evolved from the original aroma characteristics of raw materials to the mature aroma characteristics formed during fermentation. However, sensor responses may be affected by the overlapping signals of different volatile substances, making it difficult to determine the specific compounds responsible for aroma changes. Therefore, HS-SPME-GC–MS analysis was further performed to overcome the limited compound-identification capability of the electronic nose and to identify the key VOCs during fermentation.

### 3.6 HS-SPME-GC–MS analysis

Volatile organic compounds (VOCs) are key aroma-active components that determine the sensory characteristics and flavor quality of fermented vegetables (Zhu, Deng, et al., 2024). As shown in Fig. 4A and B and Table S2, a total of 76 VOCs were detected in FWBS during fermentation, including alcohols, aldehydes, esters, ketones, acids, phenols, furans, alkanes, terpenes, and other compounds. The total concentration of VOCs increased during fermentation, indicating that fermentation promoted the formation and release of aroma compounds in FWBS. Esters predominated at day 1, suggesting that early fermented samples retained fruity, sweet, and fresh aroma characteristics (Tang et al., 2022). Phenolic compounds gradually increased during fermentation and became dominant in the later stages, suggesting the development of mature fermentation characteristics (Kwaw et al., 2018). The heatmap further revealed dynamic changes in the relative abundance of VOCs during fermentation (Fig. 4C). Several esters and aldehydes, such as hexyl acetate, ethyl acetate, 2-methylbutyl 2-methylbutanoate, nonanal, and 3-methylbutanal, showed higher abundance at day 1, suggesting that the initial samples retained fresh, fruity, and plant aroma characteristics. In contrast, phenolic compounds and organic acids, including phenol, 4-vinylphenol, eugenol, 2-methoxy-4-vinylphenol, acetic acid, hexanoic acid, and octanoic acid, gradually increased during fermentation. PCA showed a clear separation among samples at different fermentation times, with no obvious overlap between groups, indicating that fermentation markedly altered the flavor profile of FWBS (Fig. 4D). Consistently, the OPLS-DA model further distinguished the samples from different fermentation stages, confirming the significant differences in VOCs during fermentation (Fig. 4E). Additionally, day 1 and day 9 both located far from the other fermentation samples, indicating distinct aroma profiles at the initial and final fermentation stages. The permutation test showed that the OPLS-DA model had high explanatory and predictive abilities, with the intercepts of the permutation regression lines were R^2^ = 0.571 and Q^2^ = −0.130 (Fig. 4F). VIP analysis further identified the key VOCs responsible for discriminating fermented samples. As shown in the VIP score plot (Fig. 4G), 15 VOCs showed VIP values greater than 1, indicating their important contributions to sample separation. Additionally, according to previously reported references, VOCs with OAV > 1 are generally considered characteristic flavor compounds (Tang et al., 2024). Therefore, VOCs with VIP > 1 and OAV > 1 were defined as key differential VOCs, and the results are summarized in Table S2. Overall, fermentation induced pronounced changes in the volatile compounds of FWBS, with the aroma gradually shifting from green and fresh notes to acidic, fruity, and ester-like characteristics. Aldehydes, esters, alcohols, and ketones were the predominant contributors to the volatile aroma characteristics of FWBS, while the 14 key differential VOCs identified by multivariate analysis may act as potential markers associated with flavor formation during fermentation. Since these changes may be closely related to microbial succession and metabolism, microbial diversity analysis was further performed to identify the key microorganisms involved in flavor formation during FWBS fermentation.

### 3.7 Metagenomic analysis

To clarify the microbial basis of flavor development, metagenomic analysis was conducted to reveal microbial community succession during FWBS fermentation. As shown in Table S3, alpha diversity analysis showed that microbial richness and diversity changed markedly during fermentation. The Goods_coverage values of all samples were close to 1.000, indicating that the sequencing depth was sufficient. Richness, Chao1, Shannon and Simpson initially decreased from day 1 to day 3, then increased and reached their highest levels on day 7, followed by a decline on day 9. The highest values of Richness, Chao1, Shannon, and Simpson were observed at day 7, suggesting that the microbial community reached its greatest complexity and diversity at this stage. β-diversity was further performed to compare differences in microbial community structure among fermentation stages. As shown in Fig. 3A, the PCoA plot showed clear separation among samples, indicating that fermentation time markedly affected microbial community structure. The first two principal coordinates explained most of the community variation, with PCoA1 and PCoA2 accounting for 66.5% and 16.4%, respectively. Day 1 was clearly separated from the other groups, suggesting that the microbial community changed rapidly after fermentation. Day 3, day 7 and day 9 samples clustered closely together, suggesting similar microbial community structures. Hierarchical clustering analysis divided the samples into three clusters, with day 1 and day 7 forming separate clusters, while day 3, day 5, and day 9 clustered together. (Fig. 3B). This pattern was characterized by progressive acidification and the enrichment of lactic acid bacteria. At the phylum level, Bacillota dominated most fermentation stages, whereas Pseudomonadota increased markedly at day 7 (Fig. 3C). This change may be driven by the dynamic fermentation environment of FWBS, including variations in nutrient availability, oxygen conditions, and microbial interactions (Tang et al., 2024). Similar microbial fluctuations have also been observed in other fermented vegetables, where LAB contribute to acidification, microbial stability, and flavor development (Chen et al., 2023). At the genus level, *Leuconostoc*, *Lactococcus*, *Weissella* and *Exiguobacterium* were dominant at the early stage (day 1- day 3). These genera are commonly detected in fermented vegetables and are associated with carbohydrate utilization, acid production and early flavor precursor formation. As fermentation proceeded, *Lactiplantibacillus* increased markedly at day 3 and day 5. At day 7, the increased abundance of *Enterococcus*, *Pediococcus* reflected a transitional community structure. By day 9, *Lactiplantibacillus*, *Leuconostoc* and *Weissella* remained the dominant genera. At the species level, *Leuconostoc citreum, Leuconostoc mesenteroides, Weissella cibaria* and *Leuconostoc inhae* were dominant at day 1 (Fig. 3E). These bacteria convert sugars into lactic acid, acetic acid, ethanol and CO₂, contributing to sourness, freshness and mild aroma in the early fermented stage. At day 3, *Lactococcus chungangensis* became the predominant species and maintained a high relative abundance until day 9, playing an important role in lactic acid production and flavor precursor formation through carbohydrate, protein and lipid metabolism. Overall, Leuconostoc, Lactococcus and Weissella mainly contributed to early fermentation (day 1 – day 3), while Lactiplantibacillus, especially Lactiplantibacillus plantarum, became dominant during the middle and late stages (day 5 – day 9). The Venn diagram showed that 2912 OTUs were shared among all fermentation stages (Fig. 3F), indicating the presence of a core microbial community throughout FWBS fermentation. The number of unique OTUs varied among stages, with day 7 showing the highest value (3962), followed by day 5 (1893) and day 1 (1606), while day 3 and day 9 had fewer unique OTUs. This pattern indicated that microbial richness increased markedly at the middle-late fermentation stage, especially at day 7, which was consistent with the alpha diversity (Table S3).

**Fig. 3:**
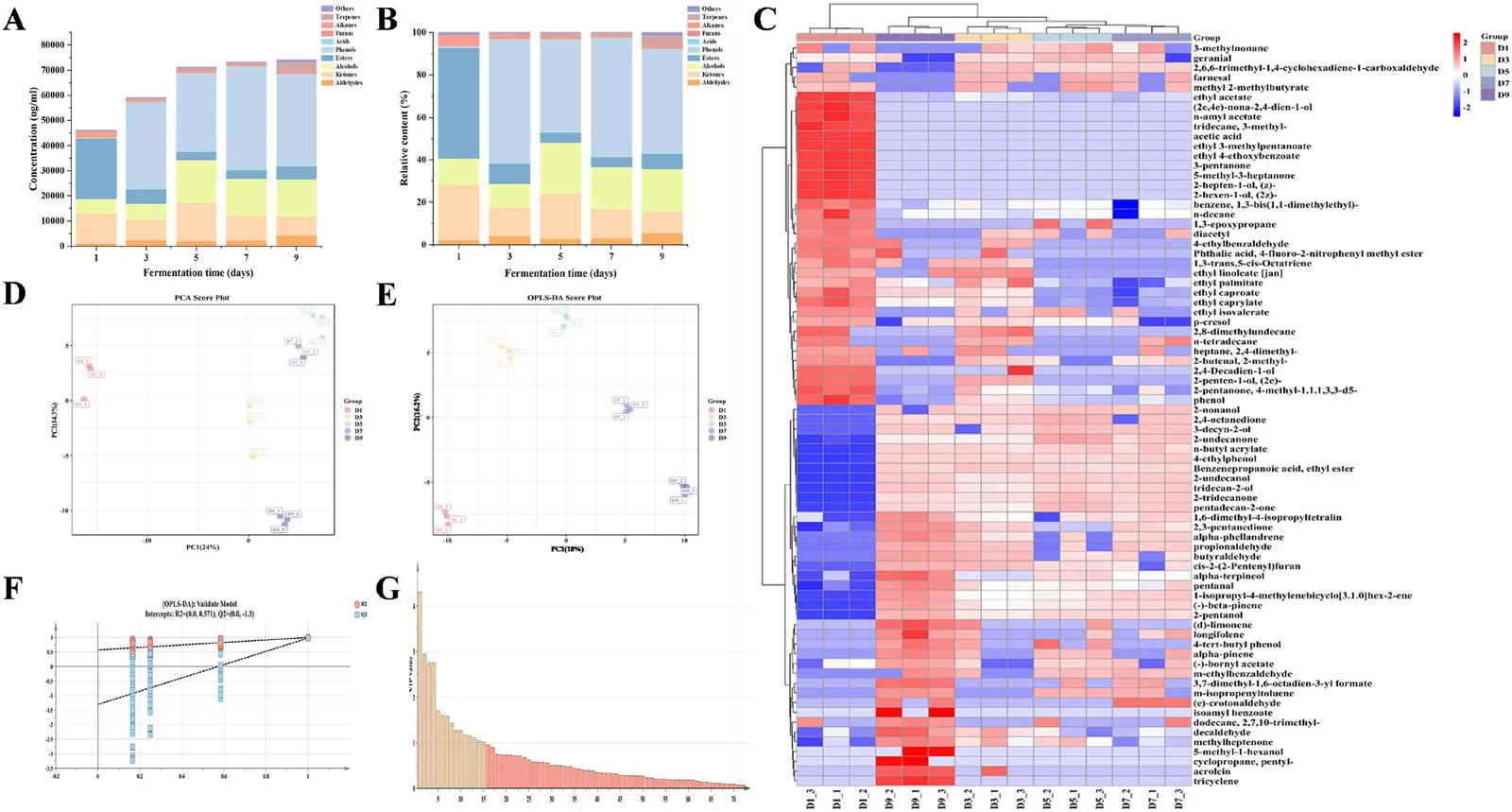
HS-SPME-GC–MS analysis of volatile organic compounds (VOCs) during water bamboo shoot fermentation. (A, B) Changes in the content and relative proportion of VOCs. (C) Heatmap showing the distribution of VOCs among samples. (D) PCA score plot. (E) OPLS-DA score plot. (F) OPLS-DA model validation. (G) VIP scores of differential VOCs. D1, D3, D5, D7 and D9 represent fermentation days 1, 3, 5, 7 and 9, respectively.

#### 3.7.1 Taxonomic composition of microbial communities

LEfSe analysis was performed to identify the discriminative microbial taxa responsible for the differences among fermentation stages. The cladogram and LDA score plot revealed stage-specific microbial biomarkers, with higher LDA scores indicating greater contributions to microbial differences among fermentation stages (Xiong et al., 2024). At the initial stage, day 1 was mainly characterized by the enrichment of p_Bacillota, c_Bacilli, o_Lactobacillales, g_*Leuconostoc*, g_*Lactococcus*, g_*Weissella*, s_*Leuconostoc citreum*, and s_*Weissella cibaria*. Day 3 was enriched with g_*Lactiplantibacillus*, suggesting that this genus became a representative biomarker during the early-to-middle fermentation stage. The enrichment of g_*Lactiplantibacillus* was consistent with the rapid accumulation of organic acids, indicating its potential role in acidification and flavor development. At day 5, o_Alteromonadales, f_Shewanellaceae, and g_*Shewanella* were significantly enriched, suggesting that the microbial community became more complex during the middle stage of fermentation. Since day 5 was also characterized by relatively high levels of total free amino acids, GABA, and lactic acid, these differential taxa may be associated with amino acid transformation, nitrogen metabolism, and flavor precursor conversion. Day 7 was mainly characterized by the enrichment of p_Pseudomonadota, c_Gammaproteobacteria, o_Moraxellales, f_Moraxellaceae, o_Pseudomonadales, g_*Acinetobacter*, g_*Pseudomonas*, and g_*Enterobacter*. In contrast, day 9 was characterized by f_Lactobacillaceae and s_*Lactiplantibacillus plantarum*, indicating that LAB contributed to the late fermentation ecosystem. Overall, the LEfSe results confirmed a stage-dependent succession of differential microbial taxa during WBS fermentation. Early fermentation was mainly associated with g_*Leuconostoc*, g_*Weissella*, and g_*Lactococcus*, whereas g_*Lactiplantibacillus* became more prominent during the middle and late stages. These findings suggest that g_*Lactiplantibacillus*, g_*Leuconostoc*, g_*Weissella*, and g_*Lactococcus* may be potential core microorganisms involved in acidification and flavor formation during FWBS fermentation.

#### 3.7.2 Microbial functional annotation

To further explore the potential metabolic functions of the microbial community during FWBS fermentation, KEGG functional annotation was performed (Fig. 4C). The predicted functions were mainly assigned to metabolism, genetic information processing, environmental information processing, cellular processes, human diseases, and organismal systems. Among these categories, metabolism was the most abundant functional category, indicating that microbial metabolic activity was the dominant functional feature during FWBS fermentation. Within the metabolism category, the major enriched pathways included carbohydrate metabolism, amino acid metabolism, energy metabolism, lipid metabolism, and metabolism of cofactors and vitamins. Carbohydrate metabolism was highly enriched throughout fermentation, indicating that microorganisms actively utilized carbohydrates in FWBS as carbon sources. This process may facilitate the production of lactic acid, succinic acid, and other OAs, thereby contributing to acidification and sour taste formation. This finding was consistent with the observed accumulation of lactic acid and succinic acid during FWBS fermentation (Table 2). Amino acid metabolism was also highly represented, suggesting that microorganisms participated in the release, transformation, and subsequent metabolism of FAAs. Combined with the changes in FAAs, microbial amino acid metabolism may contribute to the formation of umami, sweetness, bitterness, and flavor complexity. Additionally, the enrichment of lipid metabolism and energy metabolism may be associated with fatty acid degradation, energy supply, and the formation of volatile compounds such as aldehydes, ketones, acids, and esters. Meanwhile, the enrichment of cofactor and vitamin metabolism suggested that microorganisms may have the potential to participate in the biosynthesis or transformation of thiamine, riboflavin, niacin, pyridoxine, folate, biotin, pantothenic acid, and coenzyme A, potentially improving the nutritional quality of FWBS (Carrizo et al., 2016). (See Fig. 5.)

**Fig. 4:**
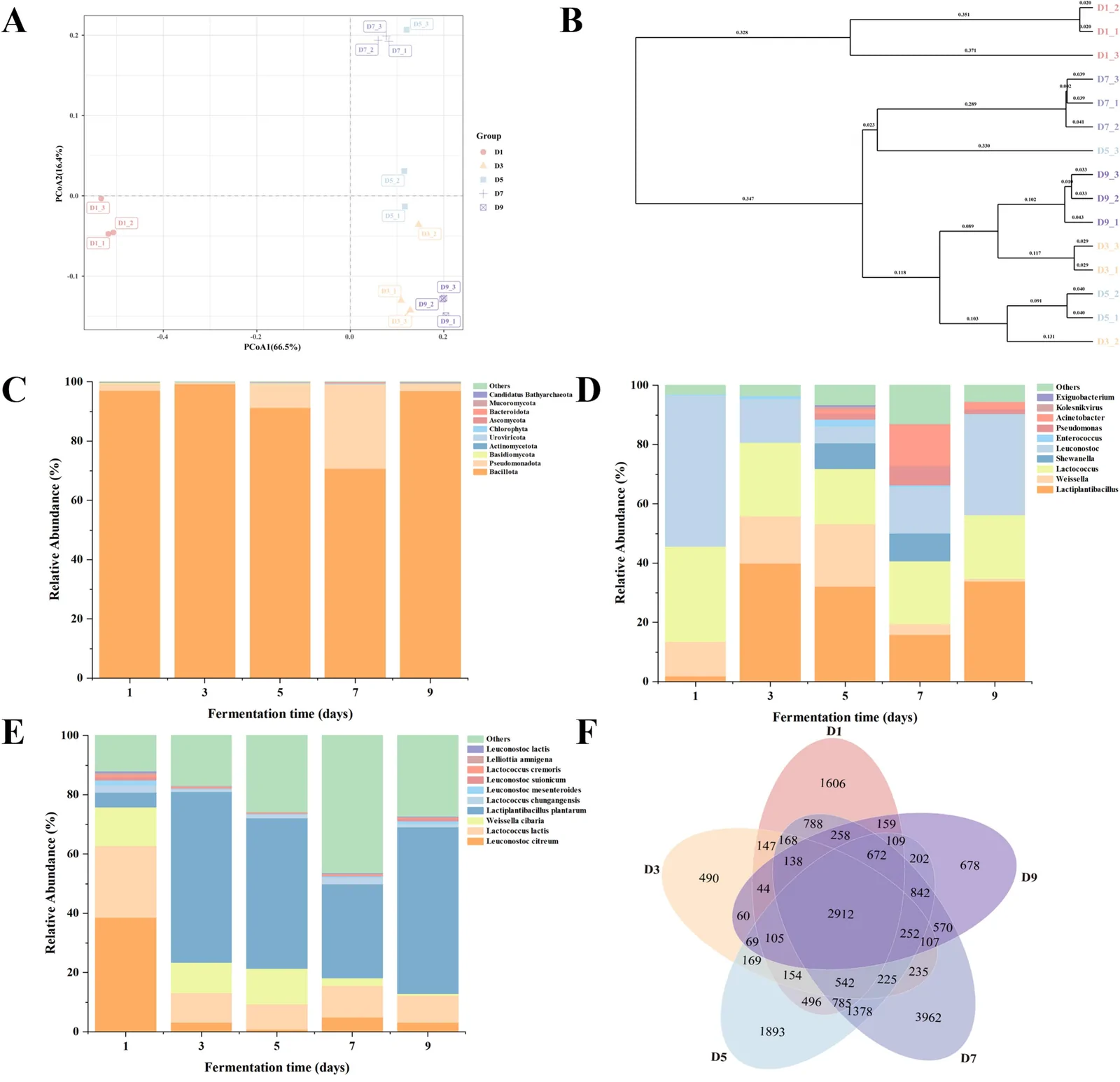
Microbial diversity and composition during water bamboo shoot fermentation. (A) Principal coordinate analysis (PCoA). (B) Hierarchical clustering analysis. Relative abundance of dominant microorganisms at the (C) phylum, (D) genus, and (E) species levels. (F) Venn diagram showing the shared and unique species among samples. D1, D3, D5, D7 and D9 represent fermentation days 1, 3, 5, 7 and 9, respectively.

**Fig. 5:**
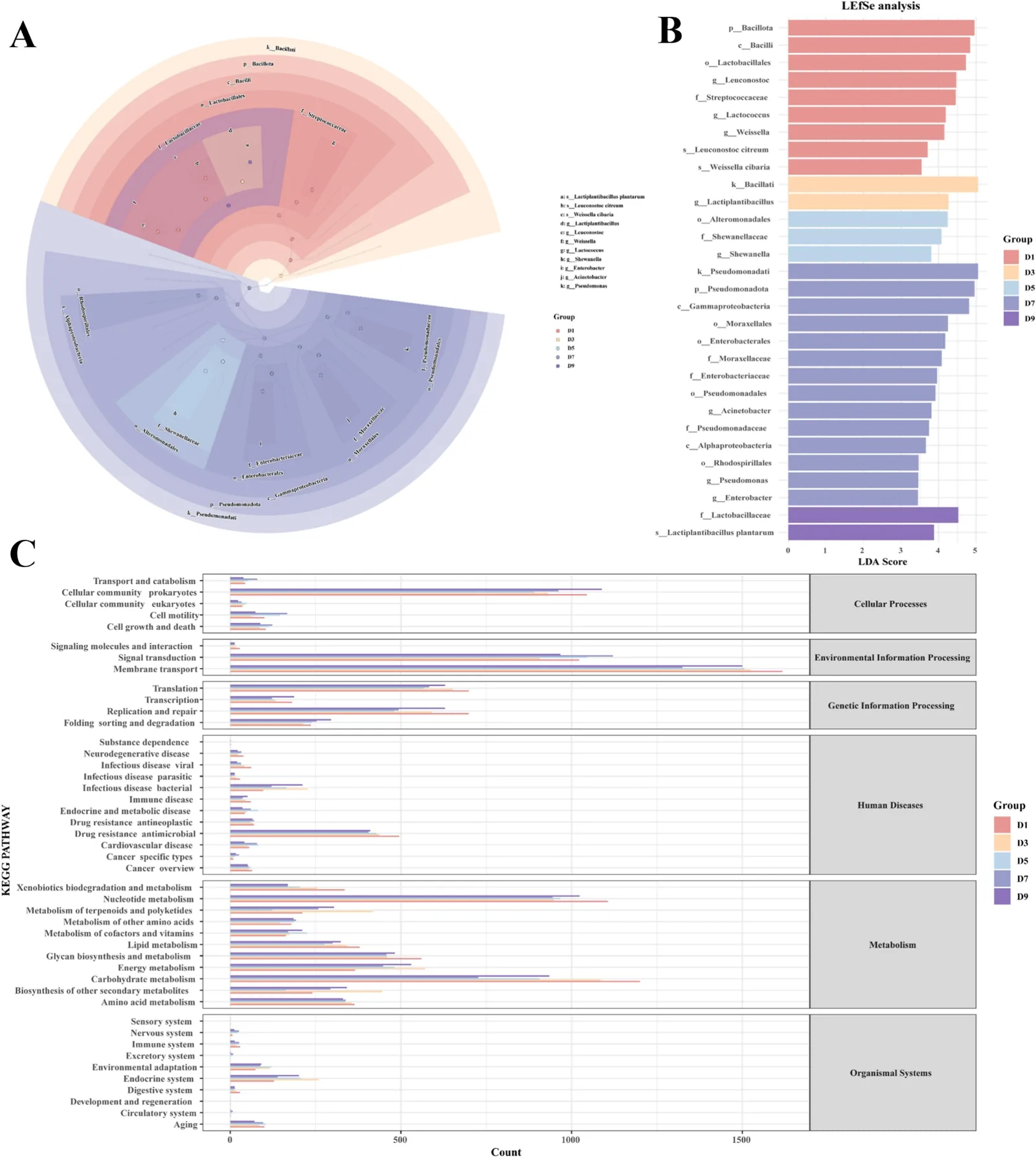
Differential microbial taxa and KEGG functional profiles during water bamboo shoot fermentation. (A) LEfSe cladogram of differential microbial taxa. (B) LDA score plot of microbial biomarkers identified by LEfSe analysis. (C) KEGG functional annotation profiles of microbial communities. The horizontal axis represents the number of annotated genes, and the vertical axis shows KEGG functional categories. D1, D3, D5, D7 and D9 represent fermentation days 1, 3, 5, 7 and 9, respectively.

Environmental information processing was mainly reflected in membrane transport and signal transduction. Membrane transport may promote the uptake of sugars, peptides, amino acids, and minerals, as well as the export of OAs. Signal transduction may help microorganisms sense and adapt to pH reduction, acid stress, osmotic changes, and nutrient limitation (Papadimitriou et al., 2016). In the category of genetic information processing, functions related to translation, replication and repair, and folding, sorting and degradation were also abundant, indicating active microbial growth, protein synthesis, and stress adaptation during fermentation. Overall, the KEGG functional prediction indicated that the microbial community during FWBS fermentation was mainly involved in carbohydrate metabolism, amino acid metabolism, energy metabolism, lipid metabolism, membrane transport, and signal transduction. These functions were closely associated with OAs accumulation, FAAs transformation, and VOCs formation. Therefore, the predicted functional profiles further support that microbial metabolic activity played a key role in acidification, flavor development, and quality formation during FWBS fermentation.

### 3.8 Integrated relationships between microbial succession and flavor formation during FWBS fermentation

To clarify the relationships between microbial succession and flavor formation, Spearman correlation analysis was conducted between dominant genera and key VOCs. As shown in Fig. 6A, *Exiguobacterium* and *Kolesnikvirus* were significantly positively correlated with 2-nonanol and 2-undecanone, whereas *Lactiplantibacillus* was significantly positively correlated with pentanal and benzenepropanoic acid ethyl ester. Previous studies have shown that *Lactiplantibacillus plantarum* can promote the formation of ethyl acetate and higher alcohols in cider, and that *Lactiplantibacillus* is closely associated with esters, alcohols, and aldehydes in fermented pickled peppers (Hu et al., 2024). In contrast, *Weissella* was significantly negatively correlated with 4-ethylphenol, (−)-β-pinene and (−)-bornyl acetate. During the early stage (D1–D3), *Leuconostoc*, *Lactococcus*, and *Weissella* predominated, while *Lactiplantibacillus* became more prominent from D3 onward. These changes coincided with rapid acidification and organic acid accumulation. Consistent with the KEGG analysis, carbohydrate metabolism was highly represented and may provide central intermediates for the formation of organic acids, ethanol, acetoin, and diacetyl. At D5, *Lactiplantibacillus* remained prominent and *Shewanella* was enriched, accompanied by relatively high levels of FAAs, GABA, and lactic acid. The concurrent enrichment of amino acid and lipid metabolism may be associated with the transformation of flavor precursors and the formation of alcohols, ketones, and aldehydes. During the late stage (D7–D9), *Acinetobacter*, *Pseudomonas*, and *Enterobacter* were enriched at D7, whereas *Lactiplantibacillus* were characteristic of D9. Meanwhile, 4-ethylphenol, α-pinene, (−)-β-pinene, and (−)-bornyl acetate generally increased during the later stages. The correlation network further visualized the interactions between microorganisms and flavor compounds (Fig. 6B). *Lactiplantibacillus*, *Lactococcus*, *Leuconostoc*, *Weissella*, *Acinetobacter*, *Pseudomonas* and *Shewanella* showed high connectivity with VOCs, suggesting their potential involvement in flavor evolution during FWBS fermentation. Meanwhile, ethyl acetate, ethyl caprylate, ethyl palmitate, 2-undecanone, pentanal, 2-nonanol, α-pinene, β-pinene and 4-ethylphenol were closely connected with several microorganisms, indicating that these compounds may be important flavor markers during FWBS fermentation. Based on KEGG pathway annotation, the possible metabolic pathways of key VOCs were reconstructed (Fig. 6C). The formation of flavor metabolites was mainly associated with carbohydrate metabolism, fatty acid metabolism, amino acid metabolism, terpenoid biosynthesis and esterification metabolism. Carbohydrate metabolism provided pyruvate as a central intermediate, which could be further converted into ethanol, acetic acid, acetoin and diacetyl. Fatty acid degradation and lipid oxidation contributed to the production of pentanal, 2-nonanol, 2-undecanol and 2-undecanone. Amino acid metabolism, especially the phenylalanine pathway, was associated with the formation of 4-ethylphenol and benzenepropanoic acid ethyl ester. Terpenoid biosynthesis was related to α-pinene, β-pinene, borneol and (−)-bornyl acetate, while esterification reactions promoted the production of ethyl acetate, ethyl caprylate and ethyl palmitate. Overall, *Lactiplantibacillus*, *Lactococcus*, *Leuconostoc*, and *Weissella* may be important contributors to flavor evolution in FWBS, and their associations with key VOCs provide potential microbial targets for flavor quality control and targeted regulation of the fermentation process.

**Fig. 6:**
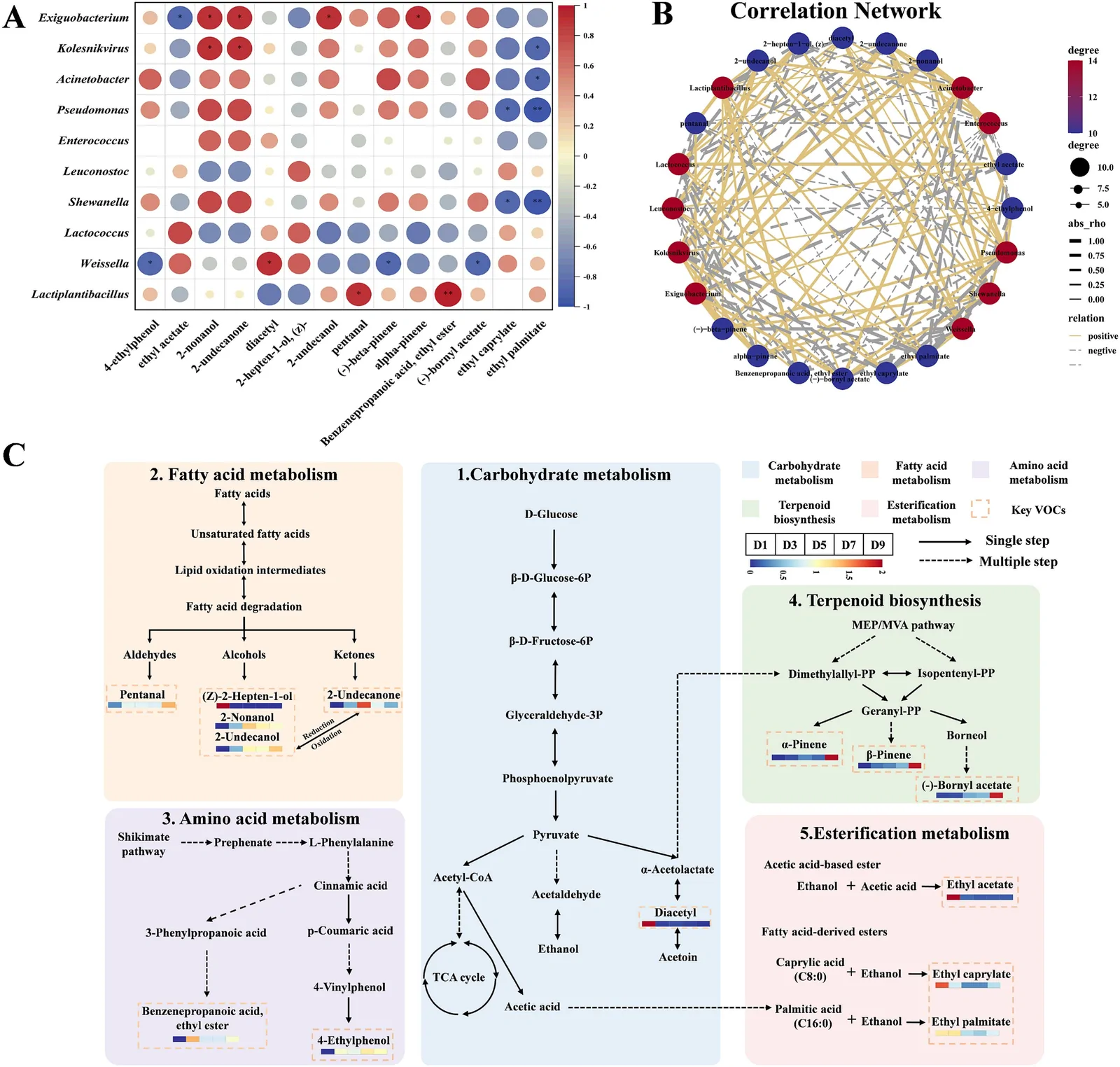
Correlation analysis between dominant microorganisms and characteristic flavor compounds and KEGG-based metabolic pathway analysis of key flavor compounds during water bamboo shoot fermentation. (A) Spearman correlation heatmap showing the associations between dominant microorganisms and key flavor compounds. Red and blue indicate positive and negative correlations, respectively, and asterisks denote statistical significance (*P < 0.05, **P < 0.01). (B) Correlation network showing the significant associations between dominant microorganisms and key flavor compounds. Nodes represent microorganisms or metabolites, and connecting lines indicate significant correlations, with different colors representing positive or negative relationships. (C) Reconstruction of metabolic pathways associated with the transformation of key flavor metabolites based on integrated metabolomic data and KEGG pathway annotation. Different colored lines represent distinct conversion routes of aroma-related compounds. D1, D3, D5, D7 and D9 represent fermentation days 1, 3, 5, 7 and 9, respectively. (For interpretation of the references to colour in this figure legend, the reader is referred to the web version of this article.)

## 4 Conclusion

In this study, the dynamic changes in flavor compounds and microbial communities during FWBS fermentation were systematically characterized. During FWBS fermentation, pH and titratable acidity showed decreasing and increasing trends, respectively, while hardness and springiness continuously declined. A total of 20 FAAs and 10 OAs were detected in FWBS, with GABA as the predominant FAA and lactic acid emerging as the major OA during fermentation. *E*-tongue and E-nose analyses distinguished FWBS samples across fermentation stages, which could be broadly divided into the early stage (day 1 – day 3), middle stage (day 5), and late stage (day 7 – day 9). HS-SPME-GC–MS detected 76 VOCs in FWBS, with esters predominating at the initial stage and phenolic compounds becoming increasingly abundant in the later stages. Additionally, 14 key differential VOCs were identified based on VIP > 1, OAV > 1, and *P* < 0.05. Mcrobial community succession was revealed, with early-stage communities mainly represented by *Leuconostoc*, *Lactococcus*, and *Weissella*, while *Lactiplantibacillus*, particularly *Lactiplantibacillus plantarum*, became more prominent during the middle and late stages. KEGG functional annotation and spearman correlation analysis further indicated that carbohydrate metabolism, amino acid metabolism, fatty acid metabolism, terpenoid biosynthesis, and esterification pathways were closely associated with the formation of 14 key VOCs. These results provide a theoretical basis for elucidating flavor formation mechanisms, screening functional starter cultures, and improving the quality control of FWBS products.

## CRediT authorship contribution statement

**Kai Zhu:** Writing – review & editing, Writing – original draft. **Songheng Wu:** Methodology, Investigation. **Huilin Deng:** Methodology, Investigation. **Chenxia Liu:** Methodology, Investigation. **Jinglin Zhang:** Methodology, Investigation. **Chao Zhu:** Resources, Methodology, Investigation. **Yongjin Qiao:** Methodology, Investigation, Funding acquisition. **Yi Zhang:** Supervision, Investigation, Funding acquisition.

## Declaration of competing interest

The authors declare that they have no known competing financial interests or personal relationships that could have appeared to influence the work reported in this paper.

## Data Availability

Data will be made available on request.

## References

[bb0005] Aoki H., Furuya Y., Endo Y., Fujimoto K. (2002). Effect of γ-aminobutyric acid-enriched tempeh-like fermented soybean (GABA-tempeh) on the blood pressure of spontaneously hypertensive rats. Bioscience, Biotechnology, and Biochemistry.

[bb0010] Asimi S., Min Z. (2025). Study on taste release and perception mechanism of japonica rice during oral processing. Food Chemistry: X.

[bb0015] Bata Gouda M.H., Peng S., Yu R., Li J., Zhao G., Chen Y., Luo H. (2021). Transcriptomics and metabolomics reveal the possible mechanism by which 1-methylcyclopropene regulates the postharvest senescence of *Zizania latifolia*. Food Quality and Safety.

[bb0020] Caffrey E.B., Sonnenburg J.L., Devkota S. (2024). Our extended microbiome: The human-relevant metabolites and biology of fermented foods. Cell Metabolism.

[bb0025] Carrizo S.L., Montes de Oca C.E., Laiño J.E., Suarez N.E., Vignolo G., LeBlanc J.G., Rollán G. (2016). Ancestral Andean grain quinoa as source of lactic acid bacteria capable to degrade phytate and produce B-group vitamins. Food Research International.

[bb0030] Chen J., Huang Y., Wang X., He J., Li W., Lu M., Yin Y. (2024). Revealing core functional microorganisms in the fermentation process of Qicaipaojiao (*Capsicum annuum* L.) based on microbial metabolic network. Food Research International.

[bb0035] Chen Q., Li Y., Yan K., Li G., Luo D., Bai W., Zeng X., Wu Q., Deng J., Dong H. (2023). Variations of volatile flavors and microbial communities in Chinese Chaozhou pickle during natural fermentation revealed by GC-IMS and high-throughput sequencing. LWT.

[bb0040] Chen Y., Wang S.W., Zhang J. (2022). Study on the application of *Zizania latifolia* straw in papermaking. BioResources.

[bb0045] Feng S., Suh J.H., Gmitter F.G., Wang Y. (2018). Differentiation between flavors of sweet orange (*Citrus sinensis*) and mandarin (*Citrus reticulata*). Journal of Agricultural and Food Chemistry.

[bb0050] Gao Y., Chen H., Liu R., Wu W., Mu H., Han Y., Gao H. (2022). Ameliorating effects of water bamboo shoot (*Zizania latifolia*) on acute alcoholism in a mice model and its chemical composition. Food Chemistry.

[bb0055] Guan Q., Huang T., Peng F., Huang J., Liu Z., Peng Z., Xie M., Xiong T. (2022). The microbial succession and their correlation with the dynamics of flavor compounds involved in the natural fermentation of suansun, a traditional Chinese fermented bamboo shoots. Food Research International.

[bb0060] Hu G., Li X., Lai A., Liu Y., Zhang Y., Wang J., Yang M. (2023). Comparative analysis of the nutritional quality of *Zizania latifolia* cultivars harvested in different growing seasons. Foods.

[bb0065] Hu K., Li J., Zhang M., Wan Y., Yang Y., Ren J., Jin Z., Wang W., Wan Y., Zhang Q., Wang D., Deng W., Zhao N., Li J., Li Q., Zhao N., Liu A., Ao X., Yang Y., Liu S. (2025). Characterizing the impact of lactic acid bacteria-fermented vegetable juice on microbial assemblage and quality of pickled suancai. Food Research International.

[bb0070] Hu L., Chen X., Cao Y., Gao P., Xu T., Xiong D., Zhao Z. (2024). *Lactiplantibacillus plantarum* exerts strain-specific effects on malolactic fermentation, antioxidant activity, and aroma profile of apple cider. Food Chemistry: X.

[bb0075] Kwaw E., Ma Y., Tchabo W., Apaliya M.T., Wu M., Sackey A.S., Tahir H.E. (2018). Effect of lactobacillus strains on phenolic profile, color attributes and antioxidant activities of lactic-acid-fermented mulberry juice. Food Chemistry.

[bb0080] Lee M., Kim D., Song J.H., Hwang I.M., Mun S.Y., Chang J.Y. (2025). Comparative insights into the metabolite and taste discrepancies upon kimchi (Mukeunji) fermentation using different *Lactobacillaceae* starters. Food Chemistry: X.

[bb0085] Leroy F., De Vuyst L. (2004). Lactic acid bacteria as functional starter cultures for the food fermentation industry. Trends in Food Science & Technology.

[bb0090] Li D., Ma X., Ren P., Zhao J., Luo A., Shi S. (2026). Dynamics and correlation of flavor compounds and bacterial communities in mustard (*Brassica juncea*) and wild jujube (*Ziziphus spinosa*) co-fermentation. Food Chemistry: X.

[bb0095] Li S., Yang F., Chen X., Duan R., Li J., Yan S. (2026). Acetic acid strengthens cell wall calcium pectate cross-linking in fresh carrot slices: Insights from pectin immunolabeling and calcium localization. Food Hydrocolloids.

[bb0105] Liao H., Luo Y., Huang X., Xia X. (2023). Dynamics of quality attributes, flavor compounds, and microbial communities during multi-driven-levels chili fermentation: Interactions between the metabolome and microbiome. Food Chemistry.

[bb0110] Liu C., Zhang J., He H., Wang Q., Wang C., Wang L., Wang X., Chen B., Zhang Y., Zhang Y., Min T., Qiao Y., Liu H. (2026). Near-freezing temperature storage delays postharvest lignification and senescence of water bamboo shoot by regulating ROS metabolism and lignin biosynthesis pathway. Postharvest Biology and Technology.

[bb0115] Liu Y., Mijiti M., Xu Z., Abulikemu B. (2024). Effect of a combination of probiotics on the flavor profiling and biogenic amines of composite fermented mutton sausages. *Food* Bioscience.

[bb0120] Luo W., Wu W., Du X., Yu Y., Wu J., Xu Y., Li L. (2023). Regulation of the nitrite, biogenic amine and flavor quality of Cantonese pickle by selected lactic acid bacteria. *Food* Bioscience.

[bb0125] Miao Y., Sun M., Huo R., Chen Y., Xie J., Dong T., Zhang M. (2025). Metagenomics and volatile metabolomics reveal microbial succession and flavor formation mechanisms during fermentation of novel pasture-style Laozao. Food Chemistry: X.

[bb0130] Ong P.K.C., Acree T.E. (1999). Similarities in the aroma chemistry of gewürztraminer variety wines and lychee (*Litchi chinensis* Sonn.). Journal of Agricultural and Food Chemistry.

[bb0135] Papadimitriou K., Alegría Á., Bron P.A., de Angelis M., Gobbetti M., Kleerebezem M., Kok J. (2016). Stress physiology of lactic acid bacteria. Microbiology and Molecular Biology Reviews.

[bb0140] Peng Q., Cheng S., Lin J., Zheng H., Xie G. (2025). Metabolic and microbial functionality during the fermentation of traditional Amaranth stems: Insights from metagenomics, flavoromics, and metabolomics. Food Chemistry.

[bb0145] Powers J.J., Quinlan M.C. (1973). Flavor detection threshold values for ethyl caprylate and phenyl ethyl alcohol and estimates of the percent population having greater sensitivity. Journal of Food Science.

[bb0150] Qi X., Ji Z., Lin C., Li S., Liu J., Kan J., Qian C. (2020). Nitric oxide alleviates lignification and softening of water bamboo (*Zizania latifolia*) shoots during postharvest storage. Food Chemistry.

[bb0155] Ruan W., Liu J., Guo H., Yang S., Niu M., Yu H., Meng X. (2026). From aroma to off-flavor: Metabolomics unveils the metabolic double-sided nature of traditional Chinese fermented foods. Food Chemistry.

[bb0160] Schnabel K.O., Belitz H.D., von Ranson C. (1988). Investigations on the structure-activity relationships of odorous substances. Part 1. Detection thresholds and odour qualities of aliphatic and alicyclic compounds containing oxygen functions. Zeitschrift für Lebensmittel-Untersuchung und -Forschung.

[bb0165] Seideneck R., Schieberle P. (2011). Comparison of the key aroma compounds in hand-squeezed and unpasteurised, commercial NFC juices prepared from Brazilian Pera Rio oranges. European Food Research and Technology.

[bb0170] Tang H., Ma J., Chen L., Jiang L., Kang L., Guo Y., Men G., Nie D., Zhong R. (2024). Characterization of key flavor substances and their microbial sources in traditional sour bamboo shoots. Food Chemistry.

[bb0175] Tang Y., Chen G., Wang D., Hu R., Li H., Liu S., Zhang Q., Ming J., Chi Y. (2022). Effects of dry-salting and brine-pickling processes on the physicochemical properties, nonvolatile flavour profiles and bacterial community during the fermentation of Chinese salted radishes. LWT.

[bb0180] Tong X., Zhu K., Wu S., Liu X., Liu C., Wang J., Zhang Y. (2025). Comparative analysis of dried water bamboo shoots using different drying methods: Physicochemical properties and flavor. Foods.

[bb0185] Van Gemert L.J. (2011).

[bb0190] Wei J., Du J., Li K., Wang Y., Yang L., Li Z. (2025). The taste of pickled bamboo shoots: Evolution of metabolites based on electronic tongue, sensory evaluation, and non-targeted metabolomics. LWT.

[bb0195] Wei J., Huang Y. (2025). Microbial function in food fermentations: Current research and future directions. Current Research in Microbial Sciences.

[bb0200] Xiong S., Xu X., Du T., Liu Q., Huang T., Ren H., Xiong T., Xie M. (2024). Organic acids drove the microbiota succession and consequently altered the flavor quality of Laotan Suancai across fermentation rounds: Insights from the microbiome and metabolome. Food Chemistry.

[bb0205] Xu M., Zhou J., Zhu P. (2021). An electronic nose system for the monitoring of water cane shoots quality with swarm clustering algorithm. Journal of Food Safety.

[bb0210] Zhang A., Yu C., Qiu H., Zhen C., Gao B., Yang C., Wang M. (2026). Synergistic metabolism of *Limosilactobacillus fermentum* and *Acetobacter pasteurianus* combined with nutrition supplementation regulating γ-aminobutyric acid biosynthesis in solid-state fermentation of cereal vinegar. Food Chemistry.

[bb0215] Zhang W., Yu X., Xin L., Xu S., Meng X. (2024). Effect of rapid fermentation on the quality of northeastern sauerkraut analyzed based on HSSPME-GC-MS and LC-MS. LWT.

[bb0220] Zhu K., Deng L., Wu J., Zhang Y., Chen J., Wu W., Liao L. (2024). Effects of different fermentation conditions on physicochemical properties, volatile compounds and antioxidant capacity of rape moss. *Food* Bioscience.

[bb0225] Zhu K., Wu W., Jiang L., Zhang Y., Wang R., Wu J., Zhou X., Liao L. (2024). Exploring correlation between microbial communities and flavor profiles during fermentation of rape moss based on metagenomics and metabolomics. *Food* Bioscience.

