# Supplementary material for: Dynamic flavor formation and microbial succession during fermentation of water bamboo shoots: Insights from volatilomics and metagenomics
Source: Food Chem X. 2026 Oct 12;39:104575. doi: 10.1016/j.fochx.2026.104575 (PMC13682018; doi:10.1016/j.fochx.2026.104575)
Supplement: Supplementary file 1 [file mmc1.docx]

# Dynamic flavor formation and microbial succession during fermentation of water bamboo shoots: Insights from volatilomics and metagenomics

**Kai Zhu****^a,b#^, Songheng Wu^a,b#^, Huilin Deng^a,b,c^, Chenxia Liu^a,b^, Jinglin Zhang^a,b^, Chao Zhu^d^, Yongjin Qiao^a,b*^, Yi Zhang^a,b*^**

^a^ Research Center for Agricultural Products Preservation and Processing, Crop Breeding & Cultivation Research Institute, Shanghai Academy of Agricultural Sciences, Shanghai 201403, China;

^b^ Key Laboratory of Germplasm Innovation and Genetic Improvement of Grain and Oil Crops (Co-construction by Ministry and Province), Ministry of Agriculture and Rural Affairs; Key Laboratory of Agricultural Genetics and Breeding of Shanghai, Crop Breeding and Cultivation Research Institute, Shanghai Academy of Agricultural Sciences; Shanghai 201403, China

^c^ Faculty of Flavour Fragrance and Cosmetics, Shanghai Institute of Technology, shanghai 201418, China

^d^ Shanghai Jinshan Pickled Vegetables Seasoning Factory Co., Ltd, Shanghai 201500, China

^#^ Kai Zhu and Songheng Wu contributed equally to this work and are considered co- first authors.

^*^Corresponding: qiaoyongjin@saas.sh.cn (Y. Qiao), zhangyi@saas.sh.cn (Y. Zhang).

**Supplementary materials**

Table S1. Main sensitive substances of PEN3 electronic nose sensors

Table S2. The concentration of volatile flavor compounds during the fermentation of water bamboo shoots

Table S3. Alpha diversity indices of microbial communities during the fermentation of water bamboo shoots

Table S4. The aroma-active compounds of water bamboo shoots for 1 days of fermentation

Table S1. Main sensitive substances of the PEN3 electronic nose sensors

| Sensor | Sensitive substances |
| --- | --- |
| W1C | Aromatic compounds, especially benzene derivatives |
| W5S | Nitrogen oxides |
| W3C | Ammonia and aromatic compounds |
| W6S | Hydrogen compounds |
| W5C | Short-chain alkanes and aromatic compounds |
| W1S | Methyl compounds and broad-range methane compounds |
| W1W | Sulfur-containing compounds and terpenes |
| W2S | Alcohols, aldehydes, and ketones |
| W2W | Sulfur-containing compounds and aromatic compounds |
| W3S | Long-chain alkanes |

Table S2. The concentration of volatile flavor compounds during the fermentation of water bamboo shoots

| **Group (No.)** | **Volatile flavor compound** | **Concentration (mg/kg)** | | | | |
| --- | --- | --- | --- | --- | --- | --- |
|  |  | **Day1** | **Day3** | **Day5** | **Day7** | **Day9** |
|  | **Aldehydes (9)** |  |  |  |  |  |
| A1 | (e)-crotonaldehyde | 0.00 ± 0.00 | 0.00 ± 0.00 | 0.00 ± 0.00 | 114.45 ± 7.28 | 58.91 ± 51.06 |
| A2 | 2,6,6-trimethyl-1,4-cyclohexadiene-1-carboxaldehyde | 64.58 ± 56.33 | 68.85 ± 5.84 | 35.52 ± 1.06 | 45.75 ± 2.85 | 0.00 ± 0.00 |
| A3 | 2-butenal, 2-methyl- | 66.43 ± 6.62 | 19.24 ± 7.85 | 3.13 ± 5.12 | 13.29 ± 5.23 | 0.00 ± 0.00 |
| A4 | 4-ethylbenzaldehyde | 116.56 ± 9.78 | 16.62 ± 14.27 | 0.00 ± 0.00 | 0.00 ± 0.00 | 30.47 ± 52.47 |
| A5 | butyraldehyde | 0.00 ± 0.00 | 31.08 ± 7.84 | 8.26 ± 7.00 | 10.61 ± 9.96 | 59.97 ± 3.26 |

| A6 | decaldehyde | 137.21 ± 37.06 | 239.11 ± 30.20 | 159.83 ± 46.19 | 165.67 ± 17.03 | 321.10 ± 43.00 | |
| --- | --- | --- | --- | --- | --- | --- | --- |
| A7 | m-ethylbenzaldehyde | 0.00 ± 0.00 | 10.21 ± 17.38 | 23.11 ± 1.97 | 27.49 ± 5.22 | 55.24 ± 50.35 | |
| A8 | pentanal | 659.66 ± 173.56 | 1863.16 ± 252.64 | 1813.33 ± 58.57 | 1769.32 ± 399.34 | 3063.52 ± 184.12 | |
| A9 | propionaldehyde | 0.00 ± 0.00 | 252.56 ± 47.05 | 34.21 ± 58.95 | 191.03 ± 42.35 | 548.29 ± 32.57 | |
|  | **Ketones (9)** |  |  |  |  |  | |
| B1 | 2,3-pentanedione | 297.11 ± 66.91 | 806.86 ± 55.51 | 461.96 ± 11.78 | 746.90 ± 46.98 | 1031.97 ± 6.50 | |
| B2 | 2,4-octanedione | 0.00 ± 0.00 | 115.35 ± 2.62 | 276.69 ± 26.09 | 99.38 ± 86.23 | 81.33 ± 10.58 | |
| B3 | 2-pentanone, 4-methyl-1,1,1,3,3-d5- | 4578.43 ± 52.65 | 4050.99 ± 177.43 | 3824.54 ± 44.75 | 3707.16 ± 296.94 | 3624.37 ± 53.28 | |
| B4 | 2-tridecanone | 0.00 ± 0.00 | 539.01 ± 38.49 | 1486.77 ± 166.96 | 1010.40 ± 215.01 | 620.91 ± 61.10 |  |
| B5 | 2-undecanone | 34.49 ± 7.00 | 2175.34 ± 84.62 | 8818.49 ± 1119.07 | 4182.45 ± 1048.96 | 2183.15 ± 203.91 |  |

(continued on next page)

Table S2 (continued)

| **Group (No.)** | **Volatile flavor compound** | **Concentration (mg/kg)** | | | | |
| --- | --- | --- | --- | --- | --- | --- |
|  |  | **Day1** | **Day3** | **Day5** | **Day7** | **Day9** |
| B6 | 3-pentanone | 662.12 ± 78.92 | 0.00 ± 0.00 | 0.00 ± 0.00 | 0.00 ± 0.00 | 0.00 ± 0.00 |
| B7 | 5-methyl-3-heptanone | 762.69 ± 166.34 | 0.00 ± 0.00 | 0.00 ± 0.00 | 0.00 ± 0.00 | 0.00 ± 0.00 |
| B8 | diacetyl | 5642.36 ± 2984.24 | 158.21 ± 147.33 | 240.35 ± 208.02 | 170.77 ± 295.48 | 0.00 ± 0.00 |
| B9 | pentadecan-2-one | 0.00 ± 0.00 | 14.25 ± 1.33 | 37.84 ± 1.96 | 26.39 ± 4.32 | 23.18 ± 3.16 |
|  | **Alcohols (11)** |  |  |  |  |  |
| C1 | (2e,4e)-nona-2,4-dien-1-ol | 51.65 ± 23.34 | 0.00 ± 0.00 | 0.00 ± 0.00 | 0.00 ± 0.00 | 0.00 ± 0.00 |
| C2 | 2,4-Decadien-1-ol | 69.50 ± 10.26 | 96.48 ± 166.80 | 0.00 ± 0.00 | 0.00 ± 0.00 | 0.00 ± 0.00 |
| C3 | 2-hepten-1-ol, (z)- | 4968.73 ± 925.18 | 0.00 ± 0.00 | 0.00 ± 0.00 | 0.00 ± 0.00 | 0.00 ± 0.00 |
| C4 | 2-hexen-1-ol, (2z)- | 498.50 ± 70.69 | 0.00 ± 0.00 | 0.00 ± 0.00 | 0.00 ± 0.00 | 0.00 ± 0.00 |
| C5 | 2-nonanol | 0.00 ± 0.00 | 4860.90 ± 35.97 | 12313.08 ± 677.22 | 10501.81 ± 1585.77 | 9320.23 ± 8076.91 |
| C6 | 2-pentanol | 0.00 ± 0.00 | 68.94 ± 4.76 | 116.53 ± 4.03 | 107.42 ± 9.25 | 295.86 ± 15.95 |
| C7 | 2-penten-1-ol, (2e)- | 185.22 ± 26.23 | 28.85 ± 2.06 | 0.00 ± 0.00 | 0.00 ± 0.00 | 0.00 ± 0.00 |
| C8 | 2-undecanol | 0.00 ± 0.00 | 1148.75 ± 78.11 | 2701.89 ± 254.52 | 2514.81 ± 324.66 | 3318.09 ± 162.92 |
| C9 | 3-decyn-2-ol | 0.00 ± 0.00 | 316.68 ± 274.12 | 1634.67 ± 125.22 | 1232.55 ± 164.30 | 1080.92 ± 71.77 |
| C10 | 5-methyl-1-hexanol | 0.00 ± 0.00 | 0.00 ± 0.00 | 0.00 ± 0.00 | 0.00 ± 0.00 | 496.28 ± 431.76 |
| C11 | tridecan-2-ol | 0.00 ± 0.00 | 136.97 ± 7.53 | 264.70 ± 33.03 | 295.21 ± 61.26 | 329.39 ± 35.71 |
|  | **Esters (14)** |  |  |  |  |  |
| D1 | (-)-bornyl acetate | 29.37 ± 28.12 | 41.39 ± 71.39 | 188.93 ± 57.89 | 222.53 ± 194.16 | 883.82 ± 212.12 |

(continued on next page)

Table S2 (continued)

| **Group (No.)** | **Volatile flavor compound** | **Concentration (mg/kg)** | | | | |
| --- | --- | --- | --- | --- | --- | --- |
|  |  | **Day1** | **Day3** | **Day5** | **Day7** | **Day9** |
| D2 | 3,7-dimethyl-1,6-octadien-3-yl formate | 0.00 ± 0.00 | 0.00 ± 0.00 | 11.91 ± 20.33 | 22.81 ± 20.05 | 162.22 ± 23.08 |
| D3 | Benzenepropanoic acid, ethyl ester | 0.00 ± 0.00 | 1029.75 ± 247.96 | 562.35 ± 47.07 | 554.74 ± 84.82 | 710.16 ± 84.44 |
| D4 | Phthalic acid, 4-fluoro-2-nitrophenyl methyl ester | 9.50 ± 3.57 | 4.94 ± 8.25 | 0.00 ± 0.00 | 0.00 ± 0.00 | 6.60 ± 11.13 |
| D5 | ethyl 4-ethoxybenzoate | 69.30 ± 13.48 | 0.00 ± 0.00 | 0.00 ± 0.00 | 0.00 ± 0.00 | 0.00 ± 0.00 |
| D6 | ethyl acetate | 18800.27 ± 1588.62 | 1656.84 ± 211.41 | 1193.25 ± 95.22 | 1253.57 ± 253.60 | 1172.88 ± 68.03 |
| D7 | ethyl caproate | 995.69 ± 161.20 | 615.67 ± 48.64 | 319.54 ± 13.26 | 308.08 ± 88.45 | 656.53 ± 4.73 |
| D8 | ethyl caprylate | 2716.79 ± 419.47 | 1214.97 ± 99.14 | 479.50 ± 49.00 | 476.16 ± 158.89 | 1072.73 ± 66.19 |
| D9 | ethyl isovalerate | 458.23 ± 10.72 | 33.46 ± 29.14 | 101.02 ± 87.69 | 27.32 ± 47.02 | 0.00 ± 0.00 |
| D10 | ethyl linoleate [jan] | 27.49 ± 2.61 | 56.16 ± 4.45 | 0.00 ± 0.00 | 0.00 ± 0.00 | 19.20 ± 32.95 |
| D11 | ethyl palmitate | 876.86 ± 68.66 | 899.03 ± 140.43 | 508.81 ± 37.86 | 425.94 ± 123.61 | 611.12 ± 94.05 |
| D12 | isoamyl benzoate | 0.00 ± 0.00 | 0.00 ± 0.00 | 0.00 ± 0.00 | 0.00 ± 0.00 | 10.47 ± 8.92 |
| D13 | n-amyl acetate | 12.90 ± 4.33 | 0.00 ± 0.00 | 0.00 ± 0.00 | 0.00 ± 0.00 | 0.00 ± 0.00 |
| D14 | n-butyl acrylate | 0.00 ± 0.00 | 25.67 ± 4.20 | 31.24 ± 3.82 | 28.52 ± 9.44 | 27.98 ± 2.12 |
|  | **Phenols (4)** |  |  |  |  |  |
| E1 | 4-ethylphenol | 135.03 ± 17.84 | 34664.51 ± 807.68 | 31367.12 ± 793.59 | 41251.54 ± 2310.09 | 36663.21 ± 1297.88 |
| E2 | 4-tert-butyl phenol | 0.00 ± 0.00 | 3.78 ± 6.24 | 17.46 ± 20.07 | 0.00 ± 0.00 | 25.42 ± 14.46 |

(continued on next page)

Table S2 (continued)

| **Group (No.)** | **Volatile flavor compound** | **Concentration (mg/kg)** | | | | |
| --- | --- | --- | --- | --- | --- | --- |
|  |  | **Day1** | **Day3** | **Day5** | **Day7** | **Day9** |
| E3 | p-cresol | 153.63 ± 9.28 | 46.66 ± 3.19 | 23.74 ± 0.79 | 13.23 ± 22.61 | 23.19 ± 19.98 |
| E4 | phenol | 170.52 ± 11.73 | 123.16 ± 4.77 | 97.94 ± 2.64 | 99.56 ± 6.24 | 87.08 ± 5.06 |
|  | **Acids (1)** |  |  |  |  |  |
| F1 | acetic acid | 11.31 ±0.35 | 0.00 ± 0.00 | 0.00 ± 0.00 | 0.00 ± 0.00 | 0.00 ± 0.00 |
|  | **Furans (1)** |  |  |  |  |  |
| G1 | cis-2-(2-Pentenyl) furan | 0.00 ± 0.00 | 119.33 ± 11.53 | 49.98 ± 2.33 | 29.46 ± 26.18 | 168.62 ± 10.18 |
|  | **Alkanes (7)** |  |  |  |  |  |
| H1 | 2,8-dimethylundecane | 9.34 ± 7.96 | 8.22 ± 4.15 | 0.00 ± 0.00 | 0.00 ± 0.00 | 0.00 ± 0.00 |
| H2 | 3-methylnonane | 12.44 ± 11.81 | 5.06 ± 4.95 | 10.40 ± 5.93 | 6.39 ± 8.48 | 0.00 ± 0.00 |
| H3 | dodecane, 2,7,10-trimethyl- | 27.69 ± 47.66 | 0.00 ± 0.00 | 35.00 ± 60.32 | 43.85 ± 75.65 | 87.65 ± 27.08 |
| H4 | heptane, 2,4-dimethyl- | 18.39 ± 0.97 | 13.83 ± 11.84 | 0.00 ± 0.00 | 6.26 ± 10.54 | 5.55 ± 9.31 |
| H5 | n-decane | 1766.57 ± 449.06 | 610.85 ± 56.75 | 659.22 ± 25.71 | 530.00 ± 316.04 | 604.10 ± 102.82 |
| H6 | n-tetradecane | 144.48 ± 125.84 | 46.09 ± 8.78 | 0.00 ± 0.00 | 63.24 ± 78.84 | 0.00 ± 0.00 |
| H7 | tridecane, 3-methyl- | 282.47 ± 130.22 | 0.00 ± 0.00 | 0.00 ± 0.00 | 0.00 ± 0.00 | 0.00 ± 0.00 |
|  | **Terpenes (13)** |  |  |  |  |  |
| I1 | (-)-beta-pinene | 0.00 ± 0.00 | 136.52 ± 73.86 | 185.46 ± 51.30 | 303.74 ± 13.76 | 1259.78 ± 322.76 |
| I2 | (d)-limonene | 0.00 ± 0.00 | 37.71 ± 65.01 | 22.13 ± 38.03 | 0.00 ± 0.00 | 534.54 ± 406.09 |
| I3 | 1,6-dimethyl-4-isopropyltetralin | 1.27 ± 1.90 | 9.13 ± 2.04 | 9.56 ± 8.28 | 16.03 ± 4.25 | 59.79 ± 22.59 |

(continued on next page)

Table S2 (continued)

| **Group (No.)** | **Volatile flavor compound** | **Concentration (mg/kg)** | | | | |
| --- | --- | --- | --- | --- | --- | --- |
|  |  | **Day1** | **Day3** | **Day5** | **Day7** | **Day9** |
| I4 | 1-isopropyl-4-methylenebicyclo [3.1.0] hex-2-ene | 0.00 ± 0.00 | 11.70 ± 1.90 | 21.94 ± 8.88 | 41.39 ± 3.04 | 124.85 ± 27.97 |
| I5 | alpha-phellandrene | 0.00 ± 0.00 | 15.45 ± 8.82 | 5.81 ± 9.76 | 16.99 ± 2.59 | 87.78 ± 28.84 |
| I6 | alpha-pinene | 0.00 ± 0.00 | 53.19 ± 91.83 | 145.99 ± 36.02 | 90.62 ± 156.65 | 1042.37 ± 341.38 |
| I7 | alpha-terpineol | 6.72 ± 4.29 | 13.75 ± 0.73 | 27.74 ± 0.95 | 18.40 ± 0.89 | 61.06 ± 11.27 |
| I8 | farnesal | 18.01 ± 15.48 | 22.78 ± 0.72 | 18.17 ± 15.62 | 15.76 ± 13.51 | 0.00 ± 0.00 |
| I9 | geranial | 72.91 ± 14.99 | 113.97 ± 4.47 | 266.58 ± 22.29 | 122.01 ± 110.60 | 50.07 ± 86.42 |
| I10 | longifolene | 0.00 ± 0.00 | 1.78 ± 2.78 | 1.28 ± 1.91 | 3.10 ± 3.19 | 27.49 ± 31.58 |
| I11 | m-isopropenyltoluene | 0.00 ± 0.00 | 0.00 ± 0.00 | 35.97 ± 1.66 | 25.62 ± 22.07 | 87.11 ± 14.93 |
| I12 | methylheptenone | 165.47 ± 55.76 | 314.22 ± 66.30 | 311.89 ± 48.50 | 200.45 ± 35.98 | 433.38 ± 14.26 |
| I13 | tricyclene | 0.00 ± 0.00 | 0.00 ± 0.00 | 0.00 ± 0.00 | 0.00 ± 0.00 | 21.72 ± 5.97 |
|  | **Others (7)** |  |  |  |  |  |
| J1 | 1,3-epoxypropane | 80.41 ± 19.11 | 0.00 ± 0.00 | 69.68 ± 60.20 | 0.00 ± 0.00 | 0.00 ± 0.00 |
| J2 | 1,3-trans,5-cis-Octatriene | 34.67 ± 17.70 | 9.45 ± 2.69 | 0.00 ± 0.00 | 0.00 ± 0.00 | 7.49 ± 6.34 |
| J3 | acrolein | 0.00 ± 0.00 | 27.83 ± 47.90 | 0.00 ± 0.00 | 0.00 ± 0.00 | 121.77 ± 11.80 |
| J4 | benzene, 1,3-bis(1,1-dimethylethyl)- | 132.80 ± 8.75 | 70.13 ± 9.12 | 70.18 ± 2.65 | 52.14 ± 25.66 | 58.24 ± 8.06 |
| J5 | cyclopropane, pentyl- | 0.00 ± 0.00 | 0.00 ± 0.00 | 0.00 ± 0.00 | 0.00 ± 0.00 | 590.27 ± 548.66 |
| J6 | ethyl 3-methylpentanoate | 67.07 ± 1.82 | 0.00 ± 0.00 | 0.00 ± 0.00 | 0.00 ± 0.00 | 0.00 ± 0.00 |
| J7 | methyl 2-methylbutyrate | 11.72 ± 1.50 | 10.78 ± 9.36 | 15.38 ± 13.25 | 9.12 ± 7.76 | 0.00 ± 0.00 |

Table S3. Alpha diversity indices of microbial communities during the fermentation of water bamboo shoots

| Fermentation time (days) | Richness | Chao1 | Shannon | Simpson | Goods_coverage |
| --- | --- | --- | --- | --- | --- |
| 1 | 5268.33 ± 597.17 | 7736.67 ± 985.41 | 2.38 ± 0.07 | 0.776 ± 0.01 | 1.000 ± 0.00 |
| 3 | 3554.67 ± 225.15 | 5606.67 ± 330.81 | 1.95 ± 0.03 | 0.634 ± 0.02 | 1.000 ± 0.00 |
| 5 | 7096.00 ± 976.56 | 9656.67 ± 1276.34 | 2.90 ± 0.50 | 0.772 ± 0.07 | 0.999 ± 0.00 |
| 7 | 9262.33 ± 140.80 | 12733.33 ± 230.94 | 3.34 ± 0.02 | 0.870 ± 0.00 | 0.999 ± 0.00 |
| 9 | 4461.33 ± 355.21 | 6396.67 ± 521.66 | 2.14 ± 0.04 | 0.655 ± 0.01 | 1.000 ± 0.00 |

Table S4. The aroma-active compounds of water bamboo shoots for 1 days of fermentation

| Compounds | Odor threshold (μg /mL) | | Odor description^d^ | | | OAV | | | | | | | | | | | |
| --- | --- | --- | --- | --- | --- | --- | --- | --- | --- | --- | --- | --- | --- | --- | --- | --- | --- |
|  |  |  |  |  |  | Day1 | | | Day3 | | | Day5 | | Day7 | | Day9 | |
| 4-ethylphenol | 0.013^a^ | | smoky, phenolic, medicinal | | | 10386.92 | | | 2666500.77 | | | 2412855.38 | | 3173195.38 | | 2820246.92 | |
| ethyl acetate | 5.000^a^ | | fruity, sweet, grape, ethereal | | | 3760.05 | | | 331.37 | | | 238.65 | | 250.71 | | 234.58 | |
| 2-nonanol | 0.058^a^ | | fatty, waxy, fruity, green, citrus-like | | | - | | | 83808.62 | | | 212294.48 | | 181065.69 | | 160693.62 | |
| 2-undecanone | 0.007^a^ | | | waxy, fruity, fatty, floral, pineapple-like | | | 4927.14 | | | 310762.86 | | | 1259784.29 | | 597492.86 | | 311878.57 |
| diacetyl | 0.001^a^ | | | buttery, creamy, dairy-like, caramel-like | | | 5642360.00 | | | 158210.00 | | | 240350.00 | | 170770.00 | | - |
| 2-hepten-1-ol, (z)- | 0.400^a^ | | | green, grassy, fatty, oily | | | 12421.83 | | | - | | | - | | - | | - |
| 2-undecanol | | 0.0086^b^ | | | waxy, fatty, clean, oily, nutty/meaty-like | | | - | | | 133575.58 | | 314173.26 | | 292419.77 | 385824.42 | |
| pentanal | | 0.012^a^ | | | fermented, bready, fruity, nutty, berry-like | | | 54971.67 | | | 155263.33 | | 151110.83 | | 147443.33 | 255293.33 | |
| (-)-β-pinene | | 4.16^a^ | | | pine-like, resinous, woody, green | | | - | | | 32.82 | | 44.58 | | 73.01 | 302.83 | |

(continued on next page)

Table S4 (continued)

| Compounds | Odor threshold (μg /mL) | Odor description^d^ | OAV | | | | |
| --- | --- | --- | --- | --- | --- | --- | --- |
|  |  |  | Day1 | Day3 | Day5 | Day7 | Day9 |
| α-pinene | 0.014^a^ | pine-like, resinous, woody, herbal, camphoraceous | - | 3799.29 | 10427.86 | 6472.86 | 74455.00 |
| Benzenepropanoic acid, ethyl ester | 0.010^a^ | floral, honey-like, sweet, fruity, | - | 643593.75 | 351468.75 | 346712.50 | 443850.00 |
| (-)-bornyl acetate | 0.075^a^ | pine-like, camphoraceous, woody, herbal | 391.6 | 551.87 | 2519.07 | 2967.07 | 11784.27 |
| ethyl caprylate | 0.150^c^ | fruity, sweet, pineapple-like, apple-like, winey -like | 18111.93 | 8099.80 | 3196.67 | 3174.40 | 7151.53 |
| ethyl palmitate | 0.700^a^ | waxy, fatty, creamy, slightly fruity | 438.43 | 449.52 | 254.41 | 212.97 | 305.56 |

^a^ Odor threshold was obtained from Van Gemert (2011).

^b^ Odor threshold was obtained from Schnabel et al. (1988).

^c^ Odor threshold was obtained from Powers & Quinlan (1973).

^d^ Odor description of each compound was obtained from <http://www.perflavorym>.

Schnabel, K. O., Belitz, H. D., & von Ranson, C. (1988). Investigations on the structure-activity relationships of odorous substances. Part 1. Detection thresholds and odour qualities of aliphatic and alicyclic compounds containing oxygen functions. *Zeitschrift für Lebensmittel-Untersuchung und -Forschung*, *187*, 215–223. <https://doi.org/10.1007/BF01043342>

Powers, J. J., & Quinlan, M. C. (1973). Flavor detection threshold values for ethyl caprylate and phenyl ethyl alcohol and estimates of the percent population having greater sensitivity. *Journal of Food Science*, *38*(4), 724–725. <https://doi.org/10.1111/j.1365-2621.1973.tb02857.x>
